# Feasibility Study on Non-Invasive Methods for Bladder Biomechanics During Voiding Using Uro-Dynamic MRI

**DOI:** 10.3390/bioengineering13091007

**Published:** 2026-08-28

**Authors:** Juan P. Gonzalez-Pereira, Evan J. Turner, Helen M. Sargeant, Wade Bushman, Alejandro Roldan-Alzate

**Affiliations:** 1 Department of Mechanical Engineering, University of Wisconsin-Madison, Madison, WI 53706, USA; ejturner4@wisc.edu; 2 Department of Radiology, University of Wisconsin-Madison, Madison, WI 53705, USA; 3 Department of Biomedical Engineering, University of Wisconsin-Madison, Madison, WI 53706, USA; hsargeant@wisc.edu; 4 Department of Urology, University of Wisconsin-Madison, Madison WI 53705, USA; bushman@urology.wisc.edu

**Keywords:** bladder, Uro-dynamic MRI, biphasic contraction, sphericity index, linear models, frequency analysis

## Abstract

**Background**: Uro-Dynamic MRI is a non-invasive protocol used to evaluate/analyze bladder contraction. Regionalized anatomical measurements and multi-variate linear regression models (MVLRM) were used to establish a methodology to evaluate bladder contraction. **Methods**: In this exploratory study, five healthy subjects and six LUTS patients underwent Uro-Dynamic MRI during voiding. Bladders were segmented at each time point during the void. Volume (*V*) and surface area (*SA*) were recorded, resulting in urinary flow rate (Q) and sphericity index (SI) curves. Regionalized anatomical measurements were tracked and linearly correlated with Q, while symptom scores from the International Prostate Symptom Score Survey (IPSS) were correlated using MVLRM. **Results**: Healthy subjects exhibited high linear correlation of contraction with Q; the opposite was observed in LUTS patients. The occurrence of ${SI}_{max}$ coincided within two time points of $Q_{max}$ for 80% of healthy subjects and 50% of LUTS patients. MVLRM identified SI, transverse and anteroposterior lengths as correlates of LUTS diagnosis and urinary frequency. **Conclusions**: This methodology revealed distinct, biphasic patterns of bladder contraction during voiding and showed associations between symptomatic behavior and anatomical measurements. This exploratory study demonstrated a comprehensive methodology to non-invasively quantify patient-specific bladder contraction patterns during voiding that could provide physicians with relevant information on symptom-specific treatment.

## 1. Introduction

Lower urinary tract symptoms (LUTS) affect more than 50% of men over the age of 50, one-third of which experience moderate to severe symptoms of diminished urinary stream and impaired bladder emptying, which are commonly attributed to prostatic obstruction [1,2,3]. Historically, benign prostatic hyperplasia (BPH) has been the focus and culprit for most, if not all, LUTS. Nevertheless, recent studies reveal that impaired bladder contractility, also called under-active bladder (UAB), is a major contributing factor [4,5]. Impaired bladder contractility is thought to occur with age but may also occur in the face of long-standing obstruction [6]. When men with LUTS are diagnosed but not treated, symptoms frequently worsen, and a significant number of them develop urinary retention with impaired kidney function, requiring catheter drainage. In fact, in lower/middle-income countries, urinary retention and associated urinary tract infection, bladder stones, and obstructive nephropathy are a major cause of renal failure and subsequent death [7]. Medical treatment of LUTS and surgery to alleviate prostatic obstruction has, on average, reduced the incidence of these complications in high-income countries and has improved quality of life for men [8]. However, there are still cases where quality of life does not improve and symptoms recur. A gap in our understanding of how and why the bladder contracts results in challenges regarding the care of men with LUTS. Existing methods to evaluate the lower urinary tract such as uro-dynamic studies (UDS) are invasive and provide an incomplete characterization of bladder contraction, lacking dynamic anatomical information.

In recent medical imaging advances, ultrasound has been used to quantify anterior bladder wall motion [9,10], bladder volumes during filling [11,12,13,14], and to calculate the post-void urine volume or post-void residual [15,16]. Recently, magnetic resonance imaging (MRI) has proven to be a useful resource to evaluate and analyze the lower urinary tract (LUT) and bladder contraction independently from prostatic diagnosis [17,18,19,20,21]. Early studies from our group used volumetric MRI to study bladder wall thickness and its changes with age [22]. Subsequently, our first approach to non-invasively assess fluid dynamics in the LUT included a combination of volumetric MR images, acquired before and after voiding, with 2D dynamic images acquired on a sagittal plane through the center of the bladder during voiding and computational fluid dynamics (CFD) [23]. Building upon that approach, a novel Uro-dynamic MRI imaging method has been developed and proposed a dynamic, volumetric imaging protocol for evaluation of LUT biomechanics during voiding [24,25,26,27]. In this study, we use Uro-Dynamic MRI to acquire images that enable comprehensive characterization of bladder biomechanics during voiding in healthy subjects and in patients with LUTS. The non-invasive framework presented here builds upon prior work by incorporating anatomical measurements that allow functional evaluation of bladder output during voiding. Specifically, we intend to establish a baseline method for bladder contraction evaluation and compare between healthy individuals and patients with LUTS. Additionally, we aim to understand how measures of regionalized bladder contraction correlate with clinical symptom scores commonly used in the diagnosis and assessment of LUTS.

## 2. Materials and Methods

### 2.1. Subject Recruitment

In this IRB-approved, HIPAA-compliant study, five healthy male subjects (37 ± 10 years of age) and six male patients previously diagnosed with LUTS (67 ± 9 years of age) between the ages of 18 and 80 were recruited. Any potential participants with contraindications to MRI, history of allergic reaction to gadolinium-based contrast, history of overt neurological disease, urinary tract infection within the last 4 weeks, or history of urinary retention were excluded. Fifteen minutes prior to the MRI scan, 1/3 of a single dose by weight of gadolinium-based contrast was hand-injected for urine enhancement. Volunteers were equipped with a condom catheter and instructed to void without straining into a 1 L urine collection bag attached to their leg while inside a 3T MRI scanner (Signa Premier, GE Healthcare, Waukesha, WI, USA) in a supine position. After the scanning session, volunteers were asked to fill out the International Prostate Score Symptom (IPSS) survey, which assesses incomplete emptying, frequency, intermittency, urgency, weak stream, straining and nocturia. Each of these symptoms is evaluated on a scale from 0 to 5, with 0 being the symptom is never experienced and 5 being the symptom is almost always experienced, yielding a final symptom score ranging from 0 to 35. This self-evaluation survey, in combination with UDS, is used clinically to diagnose LUTS.

### 2.2. Image Acquisition

A high-density flexible array coil (AIR Coil, GE Healthcare) was used for the Uro-Dynamic MRI protocol previously described by our group. This imaging protocol consists of a 3D DIfferential Subsampling with Cartesian Ordering (DISCO) acquisition sequence with a tuned flip angle to increase signal intensity from gadolinium [25,26,28]. This volumetric acquisition sequence collected a stack of 131 sagittal slices (±15 slices) with a spatial resolution of 1 mm isotropic, a slice thickness of 2 mm, a field-of-view of 25.6 cm × 25.6 cm, and a reconstruction acceleration of 2 to form a volumetric stack. Each stack was captured with an average temporal resolution of 4.12 s (±0.6 s), generating a single volumetric stack for a total of 40 time steps per void with an average scan duration of 168 s (±22.8 s).

### 2.3. 3D Model/Rendering Creation

Volumetric stacks were imported into MIMICS (Materialise, Leuven, Belgium) and analyzed using volumetric image segmentation. During segmentation, bladder volume (urine) was identified in a semi-automatic manner (automatically with manual corrections) to create 3D renderings for each time step. Each time point was segmented by two trained researchers separate and blind from one another, then revised by a radiologist, a urologist, and compared using an inter-reader variability study. Initial and final 3D renderings for all subjects were imported into 3-Matic (Materialise, Leuven, Belgium) to qualitatively and quantitatively compare bladder deformation during voiding using a spatial deformation map between these two voiding time points with the part comparison tool built into 3-Matic.

Volume (V) and surface area (SA) were recorded for each rendering and an inter-reader variability (IRV) study was performed on these variables to evaluate segmentation reproducibility using Bland–Altman plots and linear correlations. Bladder volumes were compared with a forward differences method to estimate urinary flow rate (Q) using volumetric changes divided by the time between volumes (Equation (1)). SA and V were used to analyze bladder shape by comparing each bladder rendering to an idealized sphere of the same volume (Equation (2)). This comparison yielded a sphericity index (SI) curve and allowed a volumetric evaluation of bladder contraction at every time point during the voiding effort. 2D sphericity index calculations have been used in the past, where estimated areas of the bladders in the sagittal and transverse planes taken during bladder scans have been compared [29,30]; this method has also been used in the heart to evaluate LV changes [31]. The sphericity index presented here is a 3D measurement and uses the SA of the segmented models. Due to the small number of participants, all statistical tests comparing the cohorts were Wilcoxon Rank-Sum tests with an alpha of 0.05, and *p*-values were adjusted with the Benjamini–Hochberg (false discovery rate) method.

(1) $$Flow\;Rate\left(Q_{i}\right)=\frac{V_{i}-V_{i+1}}{{Time}_{i+1}-{Time}_{i}}$$

(2) $${SI}_{i}=\frac{{SA\;Idealized\;Sphere}_{i}}{{SA\;Models}_{i}}$$

### 2.4. Anatomical Measurements

**Figure 1:**
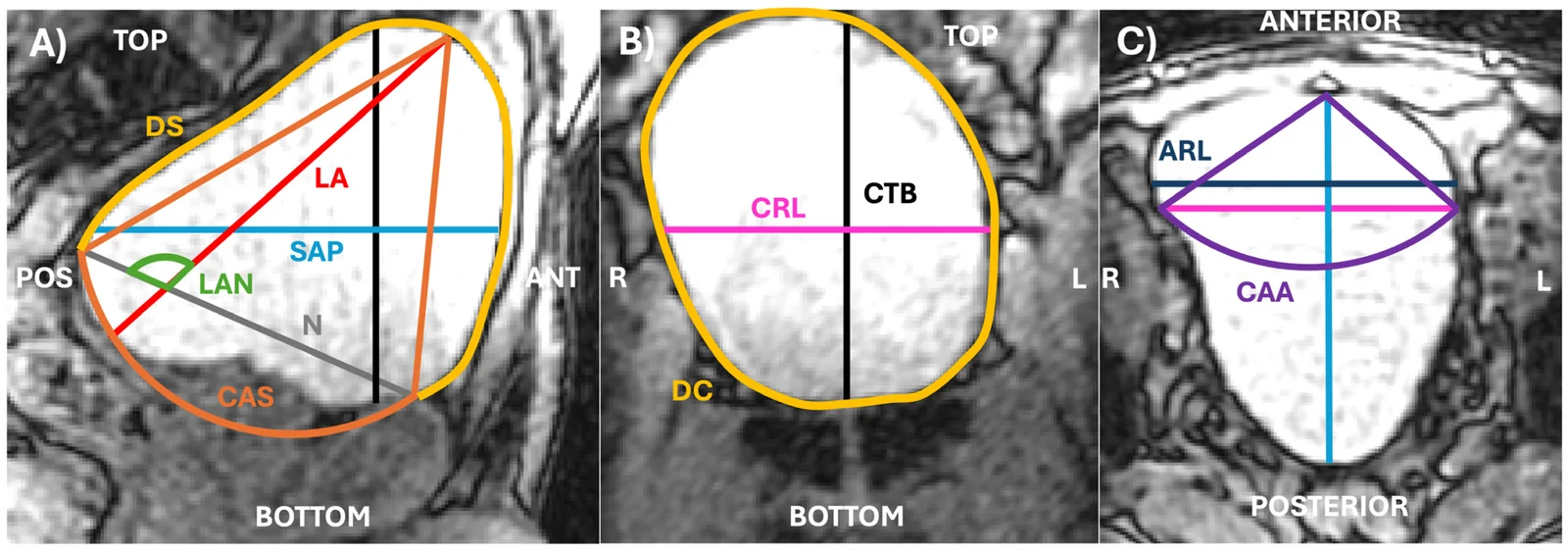
Sagittal (**A**), coronal (**B**), and axial (**C**) planes showing the anatomical measurements that were quantified and tracked during voiding for all subjects. CTB in (**A**) and SAP and CRL in (**C**) show the location and connection between the slices.

Bladder wall motion was evaluated by quantifying and tracking bladder shape using anatomical measurements in planes centered around the pubic symphysis (*PS*) and the location of insertion of the ureters at the top of the trigone for each volumetric stack. The *PS* is a consistent anatomical reference point used to standardize plane placement across subjects. The location of insertion of the ureters was used as a relative reference point during bladder voiding. Eleven measurements were identified and tracked to comprehensively quantify bladder motion observed during voiding (Figure 1); some of these have been used clinically to approximate bladder volume using the ellipsoid approximation in bladder scans. Six measurements were taken in the sagittal plane, N, LA, LAN, CAS, SAP and DS. Three measurements were taken in the coronal plane, CTB, CRL, and DC. Two measurements were taken in the axial plane, ARL, and CAA. Eight of the tracked measurements (N, LA, CTB, CRL, SAP, ARL, DS and DC) were correlated with Q; the other three were decomposed using the fast Fourier transform (FFT) [32] to quantify dominant frequency components in bladder rotation (LAN) and contraction frequencies (CAS and CAA). All the frequency-based data presented followed the Nyquist limit according to the temporal resolution for each subject. All measurements were taken in triplicate and evaluated using standard deviation and standard error of the mean to quantify methodological reproducibility for all subjects. Anatomical measurements describing transverse, caudocranial and anteroposterior deformation of the bladder were plotted against time to evaluate time dependence and were correlated with Q to evaluate regional contributions to Q.

### 2.5. Multi-Variate Linear Models

Multi-variate linear models were created to describe the relationship between the anatomical measurements, diagnosis and each reported symptom in the IPSS survey. Models were evaluated at the point of $Q_{max}$ (parameter currently used clinically for LUTS diagnosis) and the point of ${SI}_{max}$ (time point that separates bladder contraction into two phases during voiding). The proposed variables for the models were the relative time at which the event $Q_{max}$ or ${SI}_{max}$ occurs, the Q and SI at that moment, and all the tracked anatomical measurements. All variables were scaled properly, and the model was regularized using L_2_ regularization with a regularization coefficient of 0.001. Adjusted linear correlation coefficients ($R^{2}$) and significant variables with their corresponding coefficients were recorded for all created models after a Benjamini–Hochberg (false discovery rate) adjustment and *p* < 0.05. Model uncertainty and stability was calculated using the condition number, variance inflation factor (VIF), and effective degrees of freedom.

## 3. Results

### 3.1. Image Segmentation

**Figure 2:**
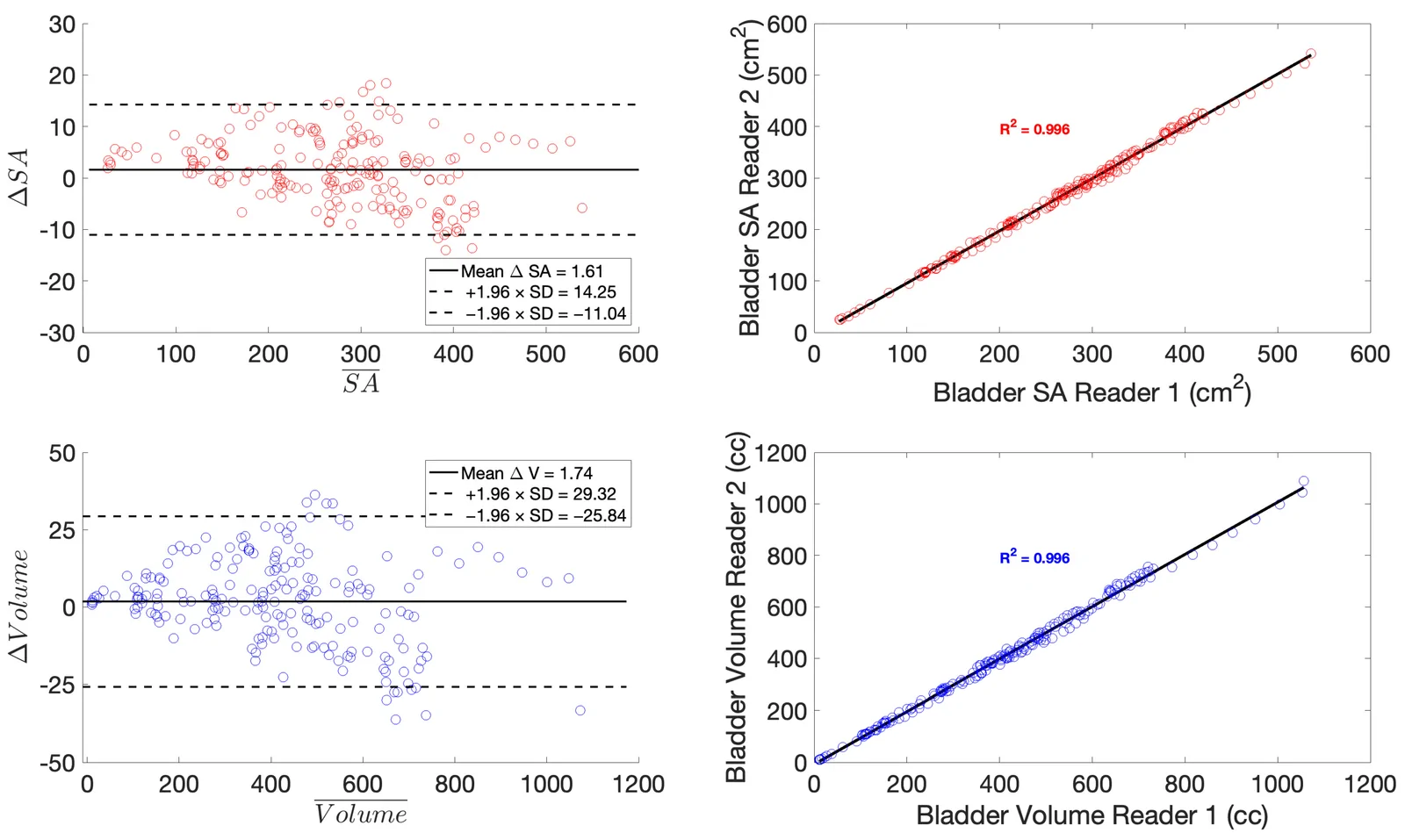
Bland–Altman plots (**left**) describing the SA (red) and V (blue) for readers 1 and 2; the constant horizontal line represents the average difference, and the dotted lines represent 95% confidence intervals. On the (**right**) are linear correlations between readers for the SA (red) and V (blue).

The combination of the Uro-Dynamic MRI imaging protocol and the analysis workflow resulted in a comprehensive approach to extract and analyze biomechanical information of the bladder during voiding using a volumetric and anatomical measurement analysis as shown in Figure 1. Bland–Altman and linear regression plots compared Vs and SAs quantified by the two readers in the inter-reader variability (IRV) study (Figure 2).

The average difference (bias) in both SA and V was close to 0, and most measurements were within the 95% confidence intervals for both variables. Measurements that fell outside of the confidence interval reported an absolute error between 5% and 8%, which agrees with previously reported errors for volume quantification using MRI [33]. Q and SI curves were derived for each subject.

Table 1 contains a summary of the results for Q and SI for each subject, and Table 2 shows average and standard deviations for each subject cohort. In the following sections, figures containing graphs, deformation maps, correlations, and frequency decompositions are provided for two characteristic subjects: Subject 001 as a healthy subject and Subject 003 as a LUTS patient. However, the same information for the rest of the subjects can be accessed in the Supplementary Materials in the corresponding sections.

**Figure 3:**
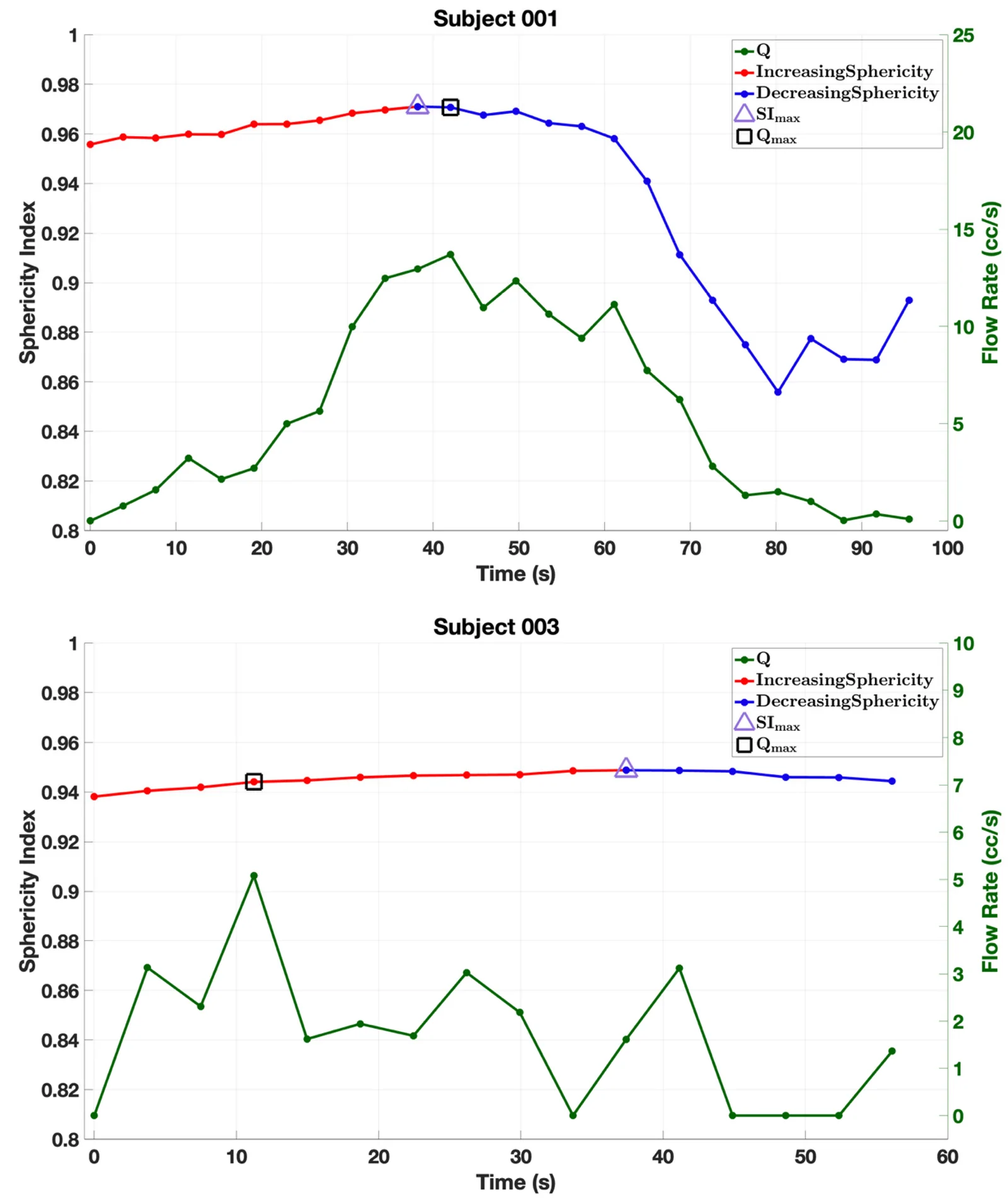
Q and SI curves over the course of the void for a healthy subject (Subject 001) and a typical LUTS subject (Subject 003). SI curve is separated into increasing (red) and decreasing (blue) at the point of ${SI}_{max}$ (purple triangle). The point of maximum flow is represented in a black square.

Q and SI, representing a healthy subject and a LUTS patient, are shown in Figure 3. Overall, Q curves showed lower $Q_{avg}$ (n.s) in LUTS patients than healthy controls throughout the void. SI increased until reaching ${SI}_{max}$, followed by a decrease until the cessation of the void for all subjects, describing a generalized biphasic behavior of bladder contraction during voiding. On average, LUTS subjects showed a lower ${SI}_{max}$ (*p*-value < 0.05) and smaller variations in SI but still showed the increasing and decreasing behavior of SI during the void. ${SI}_{max}$ was found to coincide within two time steps with $Q_{max}$ in 4 out of 5 healthy subjects and only in 3 out of 6 cases for LUTS patients. SI and Q curves, similar to the ones in Figure 3, were created for all subjects and can be seen in the Supplementary Materials S1.

**Figure 4:**
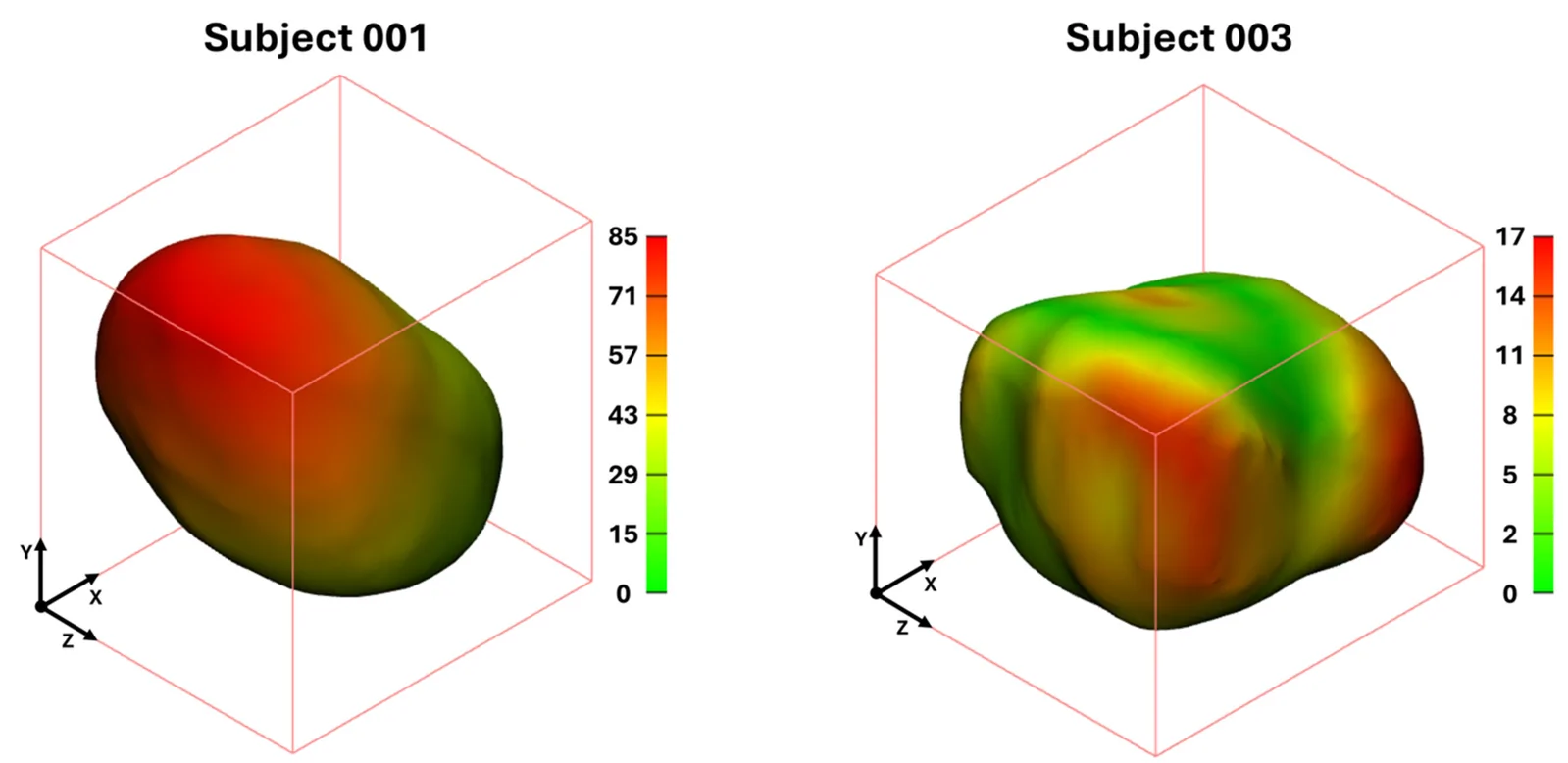
Deformation from initial to final volumes between a healthy subject (Subject 001, **left**) and a LUTS patient (Subject 003, **right**) in mm.

Deformation maps were created for all subjects using 3-Matic (Materialise, Leuven, Belgium). The comparison of these maps allowed for the observation of differences in bladder contraction between the characteristic subjects (Figure 4). Overall, healthy subjects displayed larger bladder displacements than LUTS subjects between initial and final 3D models. The deformation maps for all subjects can be observed in the Supplementary Materials S2.

### 3.2. Bladder Shape Analysis with Anatomical Measurements

**Figure 5:**
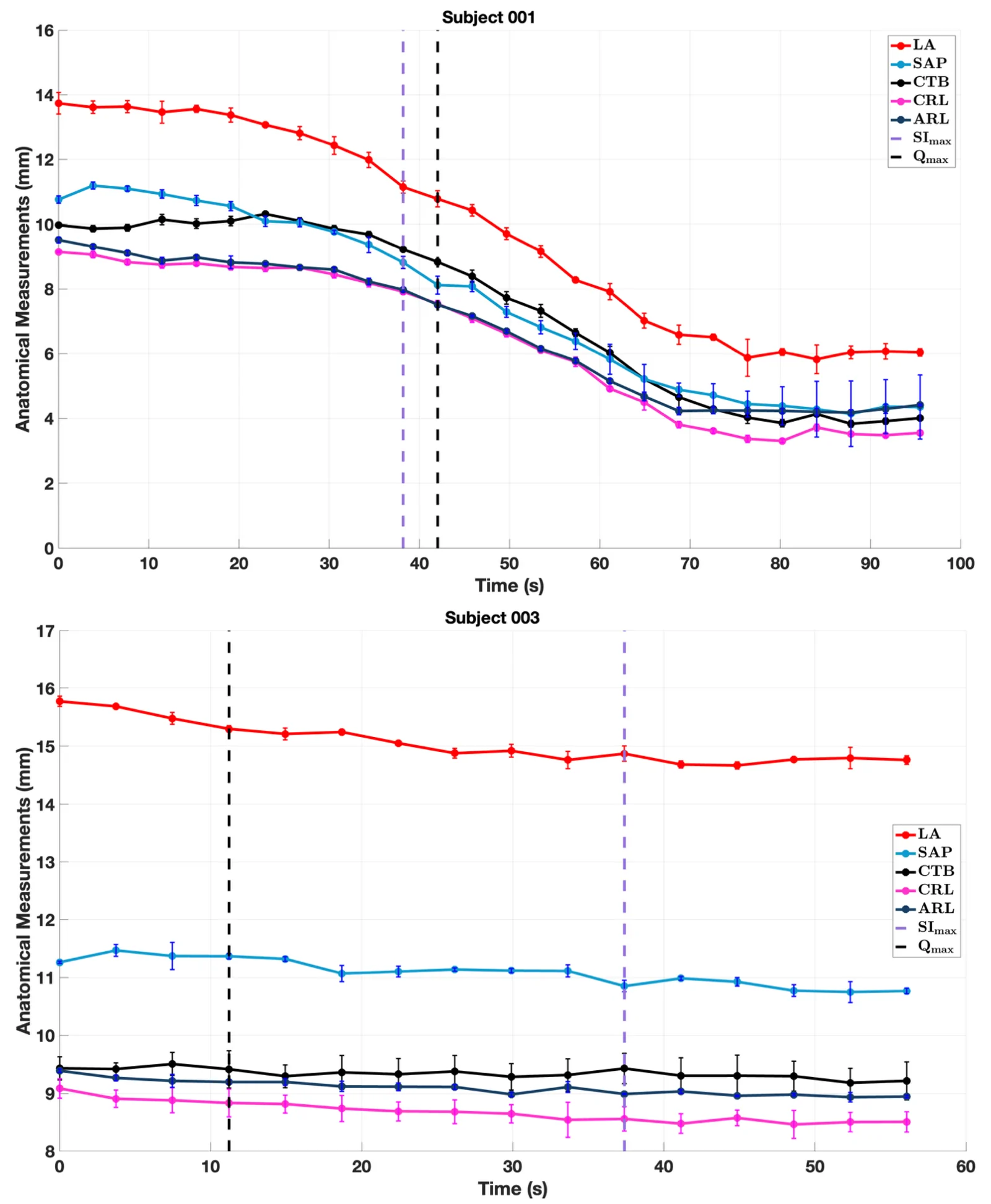
Main anatomical measurements over time for a healthy volunteer (Subject 001) and a LUTS patient (Subject 003) with vertical dotted lines representing ${SI}_{max}$ (purple) and $Q_{max}$ (black).

Anatomical measurements allowed quantification of regional contraction and bladder rotation during voiding for all subjects. Subject-specific, measurement-based analysis reported that the average difference between the measurements is smaller than 5% of the average of each measurement and is within the 95% confidence interval. Figure 5 shows the changes of select anatomical measurements with respect to time for the characteristic healthy subject and LUTS patient. To further analyze the differences in regionalized contraction, we performed correlations of anatomical measurements with Q for a functional analysis of the changes in anatomical measurements and the regionalized contributions to Q.

**Figure 6:**
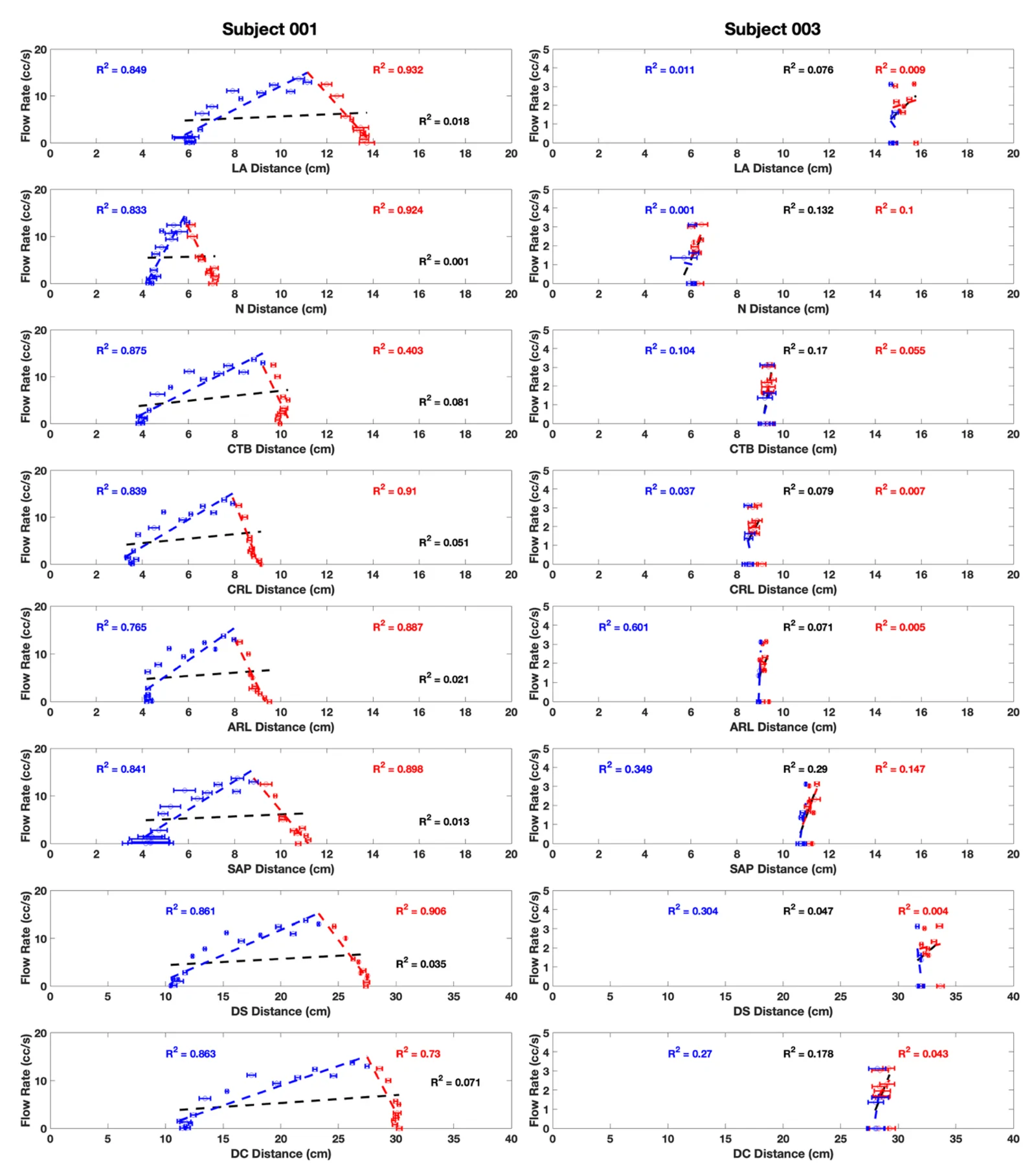
Correlations between anatomical measurements and Q for a healthy volunteer (Subject 001, **left**) and a LUTS subject (Subject 003, **right**) for the first phase (red), second phase (blue) and the full void (black) with corresponding $R^{2}$.

Figure 6 shows linear correlations of anatomical measurements (*N*, *LA*, CTB, CRL, SAP, ARL, *DS* and *DC*) with Q during the entire void for the characteristic healthy volunteer and LUTS patient. In general, the $R^{2}$ of each anatomical measurement describes the regional contribution to Q due to bladder contraction. When evaluating the void as a single-phase process, the correlation between anatomical measurements and Q was weak, as shown by low $R^{2}$ (black lines). Data sets were divided into the first and second phases, using the point of ${SI}_{max\;}$ as a phase transition point, describing increasing and decreasing sphericity (red and blue respectively). In general, subjects showed higher $R^{2}$ for most anatomical measurements when evaluating the void as a biphasic event. In the first phase, healthy subjects showed high $R^{2}$ in regions that contracted to make the bladder more spherical; however, there was variability in which region contributed more to this contraction due to pre-void differences in bladder shape and volume. In contrast, during the second phase of voiding, $R^{2}$ converged to values between 0.8 and 0.9, suggesting similar contributions to flow from most or all regions, therefore also alluding to symmetric contractions (Figure 6). Although LUTS patients showed the same biphasic contraction pattern during voiding as healthy subjects, the regionalized contributions to Q did not converge to a small range of $R^{2}$ in either phase. This analysis showed that some regions were contributing more to flow than others, suggesting asymmetric contractions, and LUTS patients also displayed an overall lack of change in dimensions throughout voiding. In general, healthy subjects showed higher $R^{2}$ than LUTS patients for all measurements in both phases of voiding. Correlations of anatomical measurements with Q for all subjects are included in the Supplementary Materials S3.

### 3.3. Rotation and Contraction Using Frequency Decomposition

**Figure 7:**
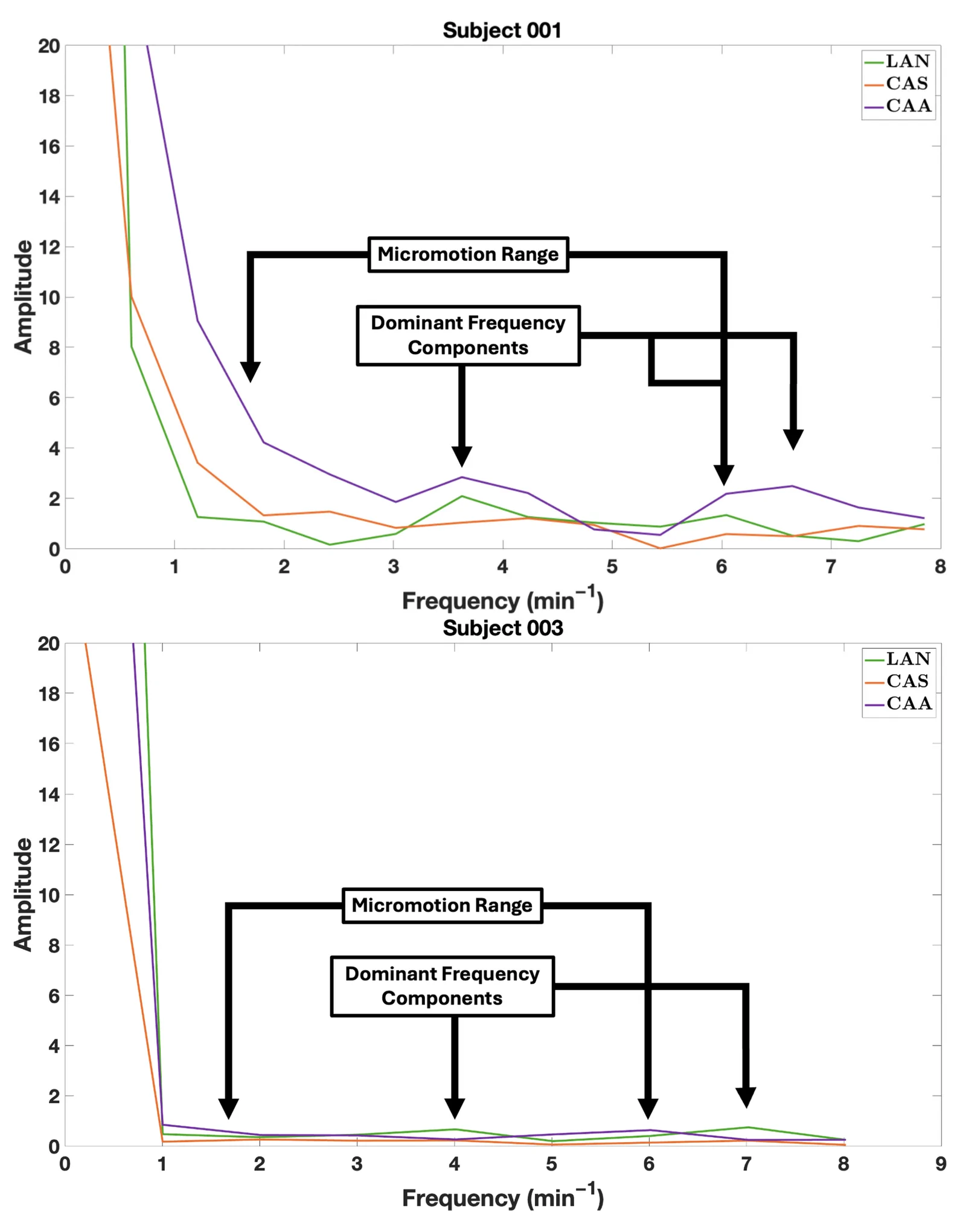
Bladder rotation (LAN, green), anterior wall contraction (CAA, purple), and dome contraction (CAS, orange) for a healthy subject (Subject 001) and a LUTS patient (Subject 003).

LAN, CAS and CAA angles were decomposed into their frequency components, yielding amplitude vs. frequency (1/minute) curves (Figure 7). Initial evaluations of all curves suggest the existence of different contraction patterns for the dome and the anterior wall of the bladder by observation of CAA and CAS measurements. Healthy subjects exhibited sparse frequency profiles for LAN, indicating marked rotation patterns with peaks from 3 to 6 rotations/min. CAS displayed a frequency profile with smaller number of peaks (n.s.) and lower maximum amplitude (n.s.), from 2 to 8 contractions/min. CAA showed frequency profiles that were less sparse due to noise components. In the case of LUTS patients, LAN exhibited frequency profiles with large amplitudes between 0 and 1 rotations/min indicating rotations around the PS at low frequencies and low amplitude peaks. CAS and CAA showed sparse frequency profiles with small peaks around 2, 4, and 7 contractions/min. Overall, healthy subjects exhibited higher amplitude dominant frequency peaks (n.s.) than LUTS patients, indicating discrete contraction and rotation patterns. Frequency decomposition graphs for all subjects are included in the Supplementary Materials S4.

### 3.4. Multi-Variate Linear Models

Ordinary least squares (OLS) models were implemented with the sub-set of variables where the outputs of the models were clinical diagnosis (0 or 1), each of the symptoms evaluated in the IPSS survey (0–5), and the summed total of the categories as the IPSS (0–35). A summary of the created models for $Q_{max}$ and ${SI}_{max}$ containing the significant variables and the corresponding coefficients are reported in Table 3 and Table 4, respectively, where significant variables were selected as the variables with *p* values < 0.05 after *p*-value adjustment with the Benjamini–Hochberg (false discovery rate). In addition, the $Q_{max}$ and ${SI}_{max}$ models had condition numbers of 18.4 and 9.8 respectively, where a value < 30 is a standard measure of global sensitivity, and the effective degrees of freedom of the $Q_{max}$ and ${SI}_{max}$ model were ~10 out of N = 33 measurements, where a value closer to the sample size would lead to instability. All the significant variables were evaluated for the VIF and showed values smaller than 5, which ensures low levels of multicollinearity [34].

Models created at the point of $Q_{max}$ displayed a range of $R^{2}$ as shown in Table 3. The models with highest correlation were diagnosis, urinary frequency, and weak stream with $R^{2}$ above 0.95. The common significant variables for these models include the bias term, age, LAN, and SI. This suggests that the behavior of the LAN in the sagittal plane and the SI provide important information that describes bladder biomechanics at the point of $Q_{max}$. SAP was an important variable, specifically in the diagnosis model, which represented deformation in the anteroposterior direction. Another important variable, specifically for the urinary frequency and weak stream models, is the CAS, which represented contraction in the dome.

Models created at the point of ${SI}_{max}$ displayed a narrower range of $R^{2}$, and good performing models had many significant variables (Table 4). The models with highest correlation were diagnosis, IPSS, urinary frequency, and weak stream, all with $R^{2}$ above 0.95. These models shared many of the same significant variables. In this case, the normalized time, or how quickly the point of ${SI}_{max}$ occurs, had a significant effect in describing diagnosis, the IPSS, and most of the symptoms. All models contain the bias term, which provides a good sense of model stability. Although almost all models include age, the correlation coefficients indicate that other variables such as SI, Q, and other anatomical measurements are similarly important for predicting symptoms. In contrast with the models trained with data obtained from the point of $Q_{max}$, the models trained from the point of ${SI}_{max}$ generally have higher correlation coefficients.

## 4. Discussion

To our knowledge, non-invasive characterization of regionalized bladder biomechanics during voiding is not possible with current diagnostic methods, where UDS is the reference standard, and fails to fully characterize voiding mechanics. This is because UDS is limited to single-point pressure measurements, 2D fluoroscopic images, and flow measurements that are taken while partially obstructing the urethra with a catheter. This study expands upon previous work [22,25,26] by involving a larger cohort of subjects, including LUTS patients, and adding regionalized evaluation of bladder contraction during voiding. In addition, the framework presented here allowed the evaluation of low-volume voids that might be deemed as invalid or incomplete voids by UDS standards.

The IRV study demonstrated high agreement between readers, low bias and an average error on the lower end of reported values for MRI segmentation [33,35,36,37], further validating the capabilities of Uro-Dynamic MRI [25,26] in terms of image acquisition quality and high reproducibility in segmentation processes. Segmented 3D renderings allowed for Q calculation and the generation of Q curves comparable to those obtained with UDS or non-invasive uroflowmetry [25,33]. SA recorded from 3D renderings was used to calculate SI curves for the whole void, unveiling a characteristic biphasic behavior of the bladder contraction for all subjects where ${SI}_{max}$ was the transition point. SI provides an overall approximation of bladder contraction pattern compared to that of a sphere of the same volume. The use of ${SI}_{max}$ as the transition point instead of $Q_{max}$ is important, as the former serves as a surrogate to understand bladder contraction from bladder shape and the latter serves as a surrogate for bladder function, allowing a separation between the bladder contracting and what is being transmitted into voiding. To our knowledge, this is the first time that a biphasic contraction pattern has been reported. The point of $Q_{max}$ was also recorded, as it is an important time during voiding for current diagnostic methods, and was contrasted against the point of ${SI}_{max}$. These key time points coincided, or nearly coincided, for most healthy subjects and for half of the LUTS patients, indicating that this could be an important metric to distinguish between cohorts and alluding to some observed symptoms to be caused by this mismatch between ${SI}_{max}$ and $Q_{max}$. Derived SI and Q curves, alongside deformation maps, revealed asymmetry in bladder contraction during voiding in LUTS patients. Healthy subjects displayed a statistically higher ${SI}_{max}$ than LUTS subjects, even though their SI remains high, which we hypothesize could be from having smaller changes in volume and a proportionally large post-void residual.

Deformation maps show symmetric contraction patterns in healthy subjects, where the dome of the bladder deforms towards the base and trigone in a decreasing gradient, with the smallest displacements at the base of the bladder. LUTS subjects show non-uniform displacements in the anterior wall of the bladder as well as asymmetric displacements in the lateral walls of the bladder. In addition, when contrasting the healthy subjects’ deformation maps with the LUTS patients’ maps, the latter show limited and asymmetric deformation patterns that suggest hypokinetic regions, or regions that did not undergo deformation, during voiding (Figure 4, and Supplementary Materials on deformation maps). These patterns have been observed in ex vivo animal models but have only been evaluated histologically and not dynamically [38]. In addition, current evaluations of hypokinetic bladder or UAB have only been evaluated with UDS or brain MRI, trying to understand signaling pathways [4,5,39,40]. To our knowledge, this is the first time that anatomical deformation maps have been used to allow the visualization of hypokinetic regions of the bladder during the voiding process. This qualitative assessment is further evaluated using anatomical measurements for region-specific behavior during voiding and should be performed in a larger cohort with clinically characterized cohorts.

Anatomical measurements were assessed over time in Figure 5. The location of ${SI}_{max}$ and $Q_{max}$ in the healthy subject suggests an inflection point in the curves, revealing regional changes before and after these points. This is not seen in the anatomical measurement curves for the LUTS subject. Preliminary results obtained from this exploratory analysis motivated an in-depth analysis that required the quantification and tracking of anatomical measurements (lengths and angles) that characterize the bladder shape. Individual analysis of all anatomical measurements for each of the two phases helped determine which region of the bladder is changing in accordance with Q at a given time during the voiding event, allowing a regionalized and non-invasive evaluation of bladder contraction. In the first phase, healthy subjects exhibited anatomical changes with rapidly increasing Q and SI, suggesting a contractile effort to become more spherical during the first phase of voiding. The second phase displayed a linear decrease in Q and anatomical measurements. The different slopes of the phase-specific linear correlation analysis allow the observation of different behaviors, or biphasic nature, of bladder contraction during voiding. For healthy subjects, *LA* was a driver of the void, as shown jointly by the deformation maps and the high $R^{2}$ with Q on both phases of voiding. The other anatomical measurements behave differently during the first phase of voiding depending on the shape of the pre-void bladder. During the second phase of voiding, healthy subjects’ anatomical measurements displayed similar contributions to Q based on $R^{2}$ convergence during this phase, suggesting a symmetric contraction of the bladder wall. LUTS patients showed multiple behaviors, which is in accordance with the large number of symptoms the diagnosis of LUTS covers [1,7,8]. Half of the LUTS patients showed similar behaviors to healthy subjects in the second phase of the void, also showing higher Q (n.s.), suggesting their cause for LUTS could have a different source than the other LUTS subjects. The results presented in this study could potentially provide valuable information to physicians for diagnosis and treatment planning after validating this methodology with a larger, age-matched and clinically characterized cohort.

Regarding the decomposed angle measurements, results in the healthy subjects show centered rotation (LAN) profiles and multiple dominant contraction peaks in CAA and CAS, indicating a more “active” bladder wall during contraction to facilitate flow. CAA and CAS do not behave consistently in all subjects, suggesting the existence of different contraction patterns for the dome and the anterior wall of the bladder. In addition, CAA displayed frequency peaks that match those observed in LAN, suggesting a connection or synchronization between anterior bladder wall contraction and rotation of the bladder during voiding. Contraction patterns focusing on the anterior bladder wall have been reported from ultrasound and show similar frequency ranges, known as the micromotion range (between 1.65 and 6 contractions/min) [10]. LUTS patients showed rotation frequency profiles with multiple low-amplitude contraction peaks and a single dominant contraction frequency. LUTS patients show similar dominant anterior and transverse contraction frequencies in CAS and CAA as healthy subjects but at much smaller amplitudes, suggesting failed or smaller contractions. Although the information provided by these measurements should be evaluated in a larger, age-matched and clinically characterized cohort, they allowed a non-invasive evaluation of healthy contraction during voiding and how these may differ from LUTS. This would be especially important to differentiate between obstructed and underactive bladders as they will require comparisons with pressure measurements.

MVLRM were used to map this complex biomechanical analysis to symptoms that are used to diagnose LUTS. These models evaluate the relationship between the different anatomical measurements and the symptom and diagnosis scores. The models were evaluated at $Q_{max}$ and ${SI}_{max}$ because the point of $Q_{max}$ is used to determine important indices for LUTS diagnosis, such as the bladder contractility index and bladder outlet obstruction index (BCI and BOOI respectively), and ${SI}_{max}$ is the point in time where the bladder changes contractile behavior. In general, the models show the importance of SI for diagnosis and symptom characterization as it reoccurs as a significant variable in the highest scoring models. In addition, the models evaluated at ${SI}_{max}$ show higher $R^{2}$, which suggests that the point of ${SI}_{max}$ provides information that could potentially aid discerning between the different types of LUTS and could allow separation between other causes for LUTS and BPH. These models have the potential to bridge complex biomechanical analysis with translational tools that are highly interpretable, as they are framed to describe symptomatic behavior, and in the future, with larger, age-matched and clinically characterized subject cohorts, predict symptoms or even diagnosis.

This framework does have some limitations, including long processing times, a small subject cohort, a lack of age-matched controls, subject orientation, limited temporal resolution, and a lack of direct and simultaneous uro-dynamic evaluation due to MRI restrictions. Temporal resolution might impact the ability to capture the exact time point and the exact value of $Q_{max}$; however, previous studies have demonstrated that Uro-Dynamic MRI protocol can reliably capture dynamic changes (i.e., Q) with the given temporal resolution [33]. Past work has shown differences in bladder shape in upright and supine positions but has resulted in no significant differences in Q [41,42]. As of right now, parallel studies are being performed to reduce the processing times and streamline the workflow; these include machine learning approaches for automatic segmentation and full Python (version 3.16) processing pipelines in local devices for easy translation into hospital machines.

## 5. Conclusions

This study demonstrates an important step towards the feasibility of MRI-based uro-dynamics in healthy subjects and in LUTS patients and sets the foundations to expand LUT biomechanical assessment. This patient-specific, non-invasive, biomechanical evaluation framework adds dynamic anatomical information to current clinical standards, bringing a new perspective to potentially aid physicians in the diagnosis and treatment of LUTS. This study presents a methodology that generated candidate imaging metrics that require validation in larger, age-matched, clinically characterized cohorts but show promise to comprehensively characterize bladder contraction non-invasively during voiding separate from prostatic evaluation and diagnosis. This framework has shown its potential by unveiling the biphasic nature of bladder contraction during voiding and by showing a regionalized, patient-specific analysis at different points during the voiding event. In addition to this biomechanical analysis, this framework outputs 3D models of the bladder and urethra that can be used for patient-specific CFD simulations to obtain pressure and velocity-derived metrics and others that cannot be calculated by UDS, such as wall shear stress [24]. Future work includes applying urodynamic MRI, in conjunction with the analysis presented here, in a larger and more diverse cohort, including subjects with a wider range of conditions such as over- and underactive bladder, neurogenic bladder, urethral strictures, etc.

## Figures and Tables

**Table 1:** Summary table for all volunteers, including measures for Q in mL/s and SI.

Subject	Condition	Age	$\bar{Q}$	$Q_{STD}$	$Q_{max}$	$\bar{SI}$	${SI}_{STD}$	${SI}_{max}$	${SI}_{min}$
Subject 1	Healthy	29	5.6	4.80	13.7	0.937	0.040	0.971	0.856
Subject 2	Healthy	55	6.18	3.33	11.24	0.957	0.012	0.969	0.931
Subject 3	LUTS	62	1.69	1.47	5.08	0.945	0.003	0.949	0.938
Subject 4	LUTS	74	1.86	2.17	6.83	0.947	0.007	0.954	0.933
Subject 5	LUTS	56	7.56	6.31	17.50	0.937	0.013	0.951	0.914
Subject 6	Healthy	34	7.30	6.94	19.41	0.954	0.006	0.962	0.943
Subject 7	Healthy	31	5.58	7.42	20.61	0.979	0.005	0.986	0.969
Subject 8	Healthy	35	5.48	2.96	11.2	0.966	0.011	0.98	0.942
Subject 9	LUTS	63	4.41	4.72	13.65	0.941	0.019	0.964	0.913
Subject 10	LUTS	79	4.41	3.68	12.44	0.915	0.003	0.92	0.908
Subject 11	LUTS	70	6.05	6.02	16.97	0.944	0.013	0.962	0.927

**Table 2:** Summary table for both cohorts, including measures for Q in mL/s and SI.

Subject Cohort	$\bar{Age}$	${Age}_{STD}$	$\bar{Q}$	$\bar{Q_{STD}}$	$\bar{Q_{max}}$	$Q_{max\;STD}$	$\bar{SI}$	$\bar{{SI}_{STD}}$	$\bar{{SI}_{max}}$	${SI}_{max\;STD}$	$\bar{{SI}_{min}}$	${SI}_{min\;STD}$
Healthy	37	10	6.03	5.09	15.23	4.50	0.959	0.015	0.974	0.0096	0.9283	0.0428
LUTS	67	9	4.33	4.06	12.08	5.15	0.938	0.0096	0.95	0.0166	0.9222	0.0120

**Table 3:** Summary table for all the models evaluated at $Q_{max}$.

Linear Models at $Q_{max}$			
Descriptor	$R^{2}$	Significant Variables	Metric Coefficients
Diagnosis	0.960	Bias, Age, Normalized Time, SAP, ARL, SI	0.454, −0.312, 0.159, −0.312, 0.455, 0.115
IPSS Score	0.908	Bias, Age, LAN, SI	12.624, 11.001, 6.360, 4.702
Incomplete Emptying	0.638	Bias, LAN	0.999, 1.070
Frequency	0.991	Bias, Age, Normalized Time, LAN, CAS, ARL, SI, Q	3.270, 1.145, −0.293, 0.340, 0.359, −0.738, −0.290, 0.133
Intermittency	0.846	Bias, Age, LAN	1.907, 1.222, 0.775
Urgency	0.668	Bias, Age, Normalized Time, LAN, SAP, Q	1.998, 1.165, −1.177, 1.620, −1.370, 1.021
Weak Stream	0.977	Bias, Age, Normalized Time, CAS, CRL, SI, Q	1.635, 2.780, 0.973, −0.552, 0.265, 1.813, 0.563
Straining	0.917	Bias, Age, LAN, CAS, CRL, SI	0.908, 2.003, 1.176, −0.670, −0.427, 1.695
Nocturia	0.849	Bias, Age, LAN, CAS, SAP, ARL, SI, Q	1.907, 2.074, 1.483, −0.607, −0.783, 0.998, 1.396, 0.358

**Table 4:** Summary table for all the models evaluated at ${SI}_{max}$.

Linear Models at ${SI}_{max}$			
Descriptor	$R^{2}$	Significant Variables	Metric Coefficients
Diagnosis	0.953	Bias, Age, Normalized Time, CAS, CRL, ARL, *SI*, *Q*	0.454, −0.300, −0.133, −0.208, 0.118, 0.179, 0.189, 0.133
IPSS Score	0.961	Bias, Age, Normalized Time, LAN, SAP, ARL, *SI*	12.624, 7.368, −2.207, 2.340, 2.502, −2.995, 2.550
Incomplete Emptying	0.645	Bias	0.999
Frequency	0.987	Bias, Age, Normalized Time, LAN, CAS, CRL, ARL, *SI, Q*	3.269, 0.714, −0.187, 0.196, 0.498, −0.362, −0.538, −0.547, 0.531
Intermittency	0.942	Bias, Age, Normalized Time, LAN	1.907, 1.245, 0.859, 0.596
Urgency	0.892	Bias, Age, Normalized Time, *Q*	1.998, 0.903, −0.601, 1.246
Weak Stream	0.973	Bias, Age, Normalized Time, LAN, CAS, CRL, SAP, ARL, *SI*	1.635, 2.062, −0.810, −0.321, −0.353, 0.347, 0.840, −1.114, 1.650
Straining	0.939	Bias, Age, Normalized Time, LAN, CAS, CRL, SAP, *SI*	0.908, 0.997, −0.590, 0.721, −0.590, −0.662, 0.697, 1.156
Nocturia	0.934	Bias, Age, Normalized Time, LAN, CAS, CRL, SAP, *SI*	1.907, 1.046, −1.003, 0.726, −0.767, −0.498, 0.620, 1.054

## Data Availability

The data presented in this study are available on request from the corresponding author due to patient privacy.

## Supplementary Materials

The following supporting information can be downloaded at https://www.mdpi.com/article/10.3390/bioengineering13091007/s1, Supplementary Materials S1: Flow Rate and Sphericity Index Curves; Supplementary Materials S2: Deformation Maps; Supplementary Materials S3: Anatomical Measurements Correlated with Flow Rate; Supplementary Materials S4: Rotation and Contraction; Frequency Decompositions.

## Abbreviations

The following abbreviations are used in this manuscript:

V	Bladder volume
SA	Bladder surface area
BPH	Benign prostatic hyperplasia
LUTS	Lower urinary tract symptoms
UDS	Multichannel uro-dynamic studies
MRI	Magnetic Resonance Imaging
MVLRM	Multi-variate linear regression models
SI	Sphericity Index
${SI}_{max}$	Maximum sphericity index
${SI}_{max\;STD}$	Standard deviation of the maximum sphericity index
${SI}_{min}$	Minimum sphericity index
${SI}_{min\;STD}$	Standard deviation of the minimum sphericity index
$\bar{SI}$	Average sphericity index
$\bar{{SI}_{STD}}$	Average standard deviation for sphericity indices
$\bar{{SI}_{max}}$	Average maximum sphericity index
$\bar{{SI}_{min}}$	Average minimum sphericity index
Q	Urinary flow rate
$Q_{max}$	Maximum urinary flow rate
$Q_{max\;STD}$	Standard deviation of the maximum urinary flow rates
$\bar{Q}$	Average urinary flow rate
$\bar{Q_{max}}$	Average maximum urinary flow rate
$\bar{Q_{STD}}$	Average standard deviation for urinary flow rates
IPSS	International prostate symptom score
LUT	Lower urinary tract
UAB	Under-active bladder
CFD	Computational fluid dynamics
IRB	Internal review board
HIPPA	Health insurance portability and accountability act
DISCO	Differential subsampling with cartesian ordering
IRV	Inter-reader variability
PS	Pubic symphysis
N	Distance between the location of ureter insertion and the top of the trigone in a sagittal plane centered in the PS
LA	Longest axis of the bladder in a sagittal plane centered in the PS
LAN	Angle between N and LA in a sagittal plane centered in the PS
CAS	Angle between both ends of N centered in the furthest end of LA in a sagittal plane centered in the PS
DS	Spline length of the dome of the bladder in a sagittal plane centered in the PS
CTB	Caudocranial length in a coronal plane centered in the middle of the PS
CRL	Transverse length in a coronal plane centered in the middle of the PS
DC	Circumference of the bladder in a coronal plane centered in the middle of the PS
SAP	Anteroposterior length in an axial plane at the height of CRL
ARL	Maximum transverse length in an axial plane at the height of CRL
CAA	Angle between both ends of CRL centered in the anterior location of SAP in an axial plane at the height of CRL
FFT	Fast Fourier transform
$R^{2}$	Linear correlation coefficient
OLS	Ordinary least squares
