# Supplementary material for: Feasibility Study on Non-Invasive Methods for Bladder Biomechanics During Voiding Using Uro-Dynamic MRI
Source: Bioengineering (Basel). 2026 Aug 28;13(9):1007. doi: 10.3390/bioengineering13091007 (PMC13603564; doi:10.3390/bioengineering13091007)
Supplement: Supplementary file 1 [file bioengineering-13-01007-s001.zip › bioengineering-4496213-supplementary.pdf]

Supplemental Document

Supplementary Materials S1: Flow Rate and Sphericity Index Curves

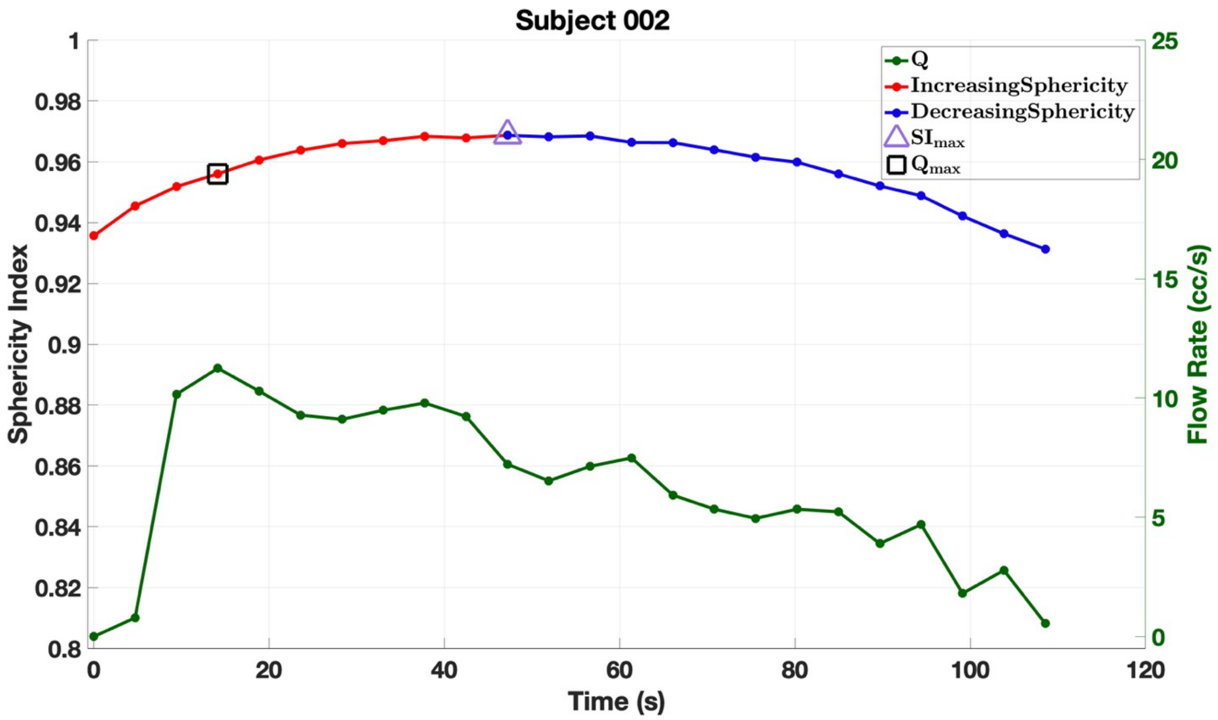

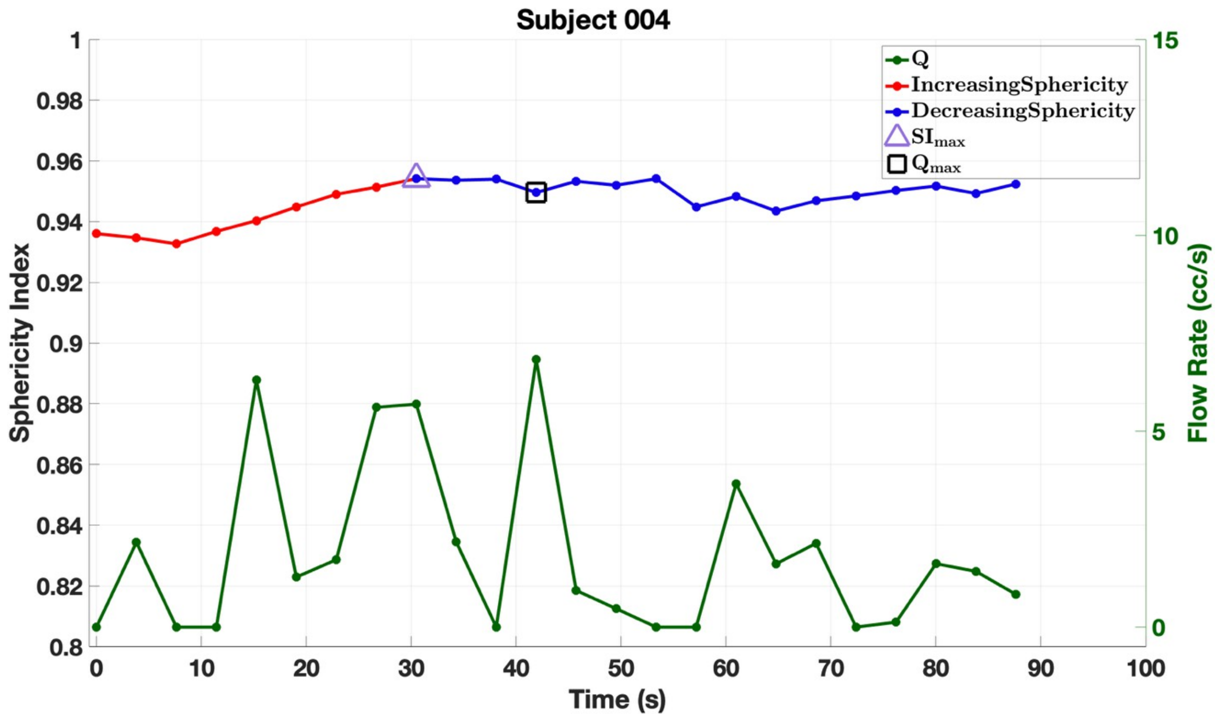

Subject 004
Sphericity Index
Flow Rate (cc/s)
Time (s)
Q
IncreasingSphericity
DecreasingSphericity
SI_{max}
Q_{max}

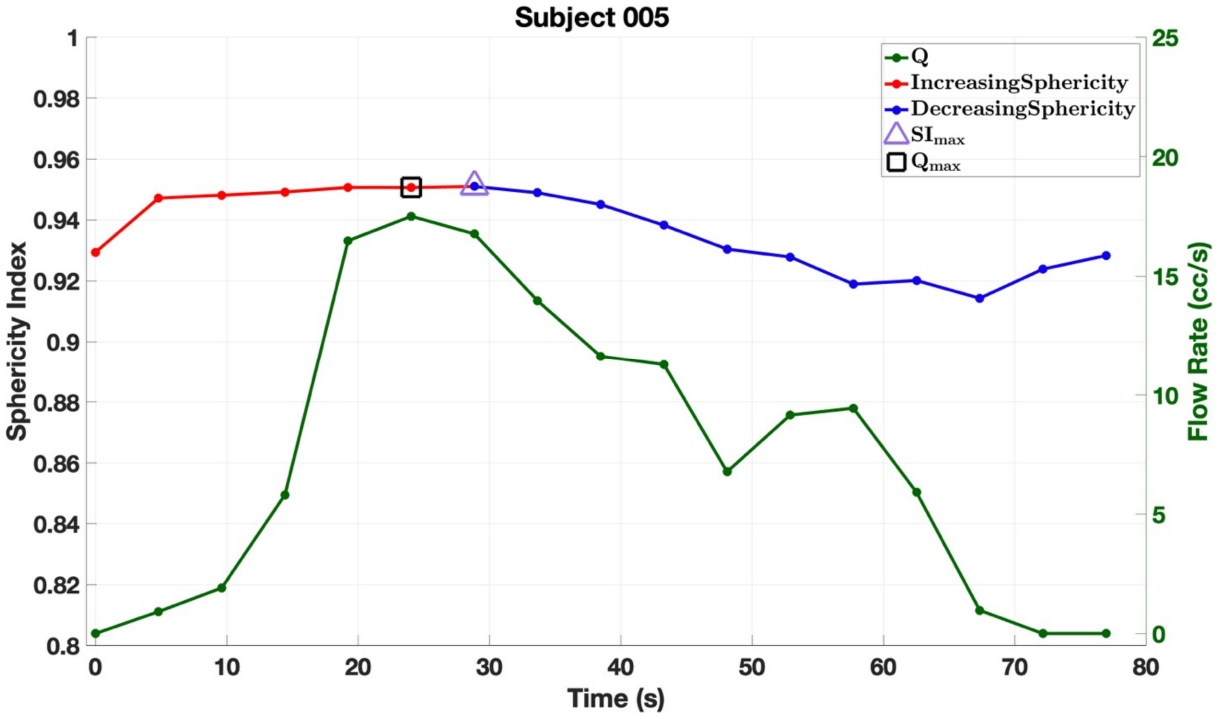

Subject 005
Sphericity Index
Flow Rate (cc/s)
Time (s)
Q
IncreasingSphericity
DecreasingSphericity
SI_{max}
Q_{max}

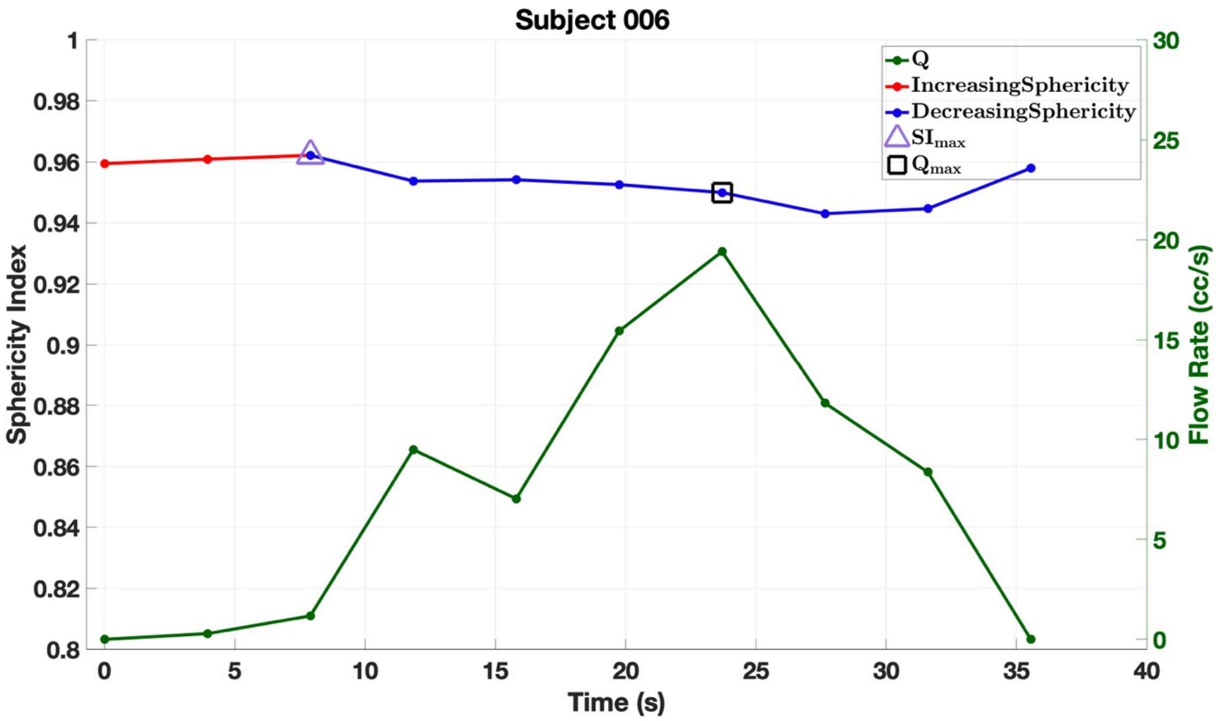
Subject 006
Sphericity Index
Flow Rate (cc/s)
Time (s)
Q
IncreasingSphericity
DecreasingSphericity
SI_max
Q_max

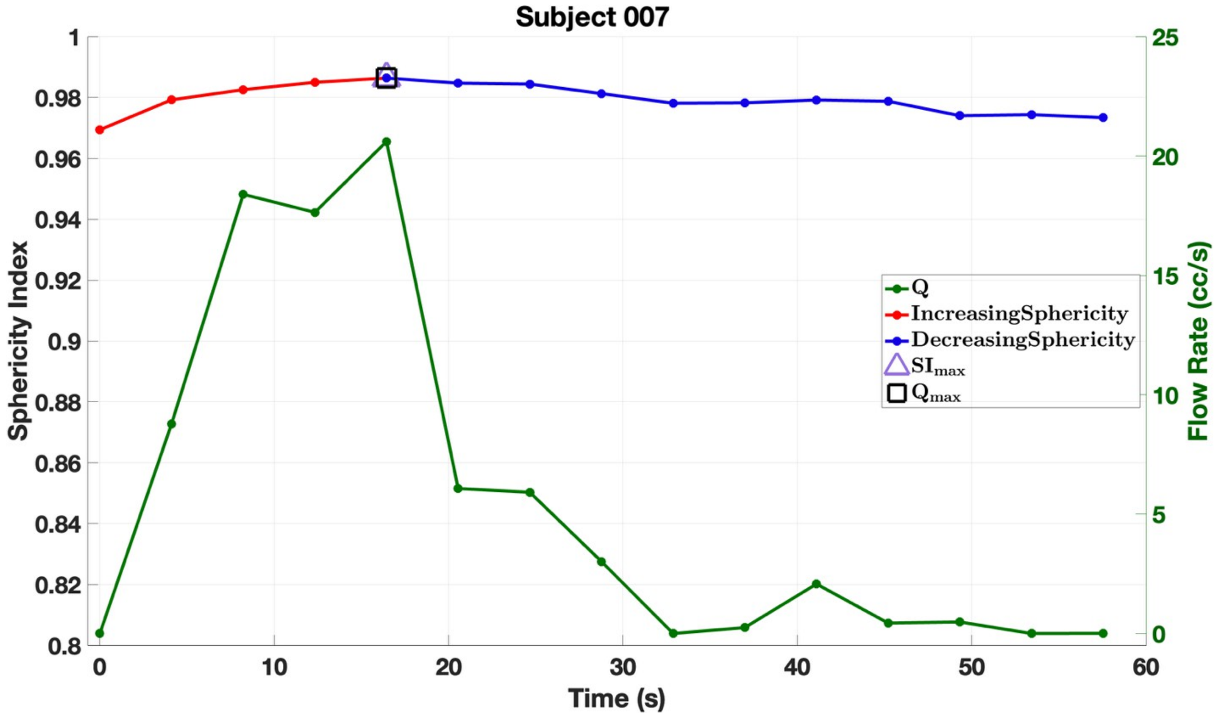
Subject 007
Sphericity Index
Flow Rate (cc/s)
Time (s)
Q
IncreasingSphericity
DecreasingSphericity
SI_max
Q_max

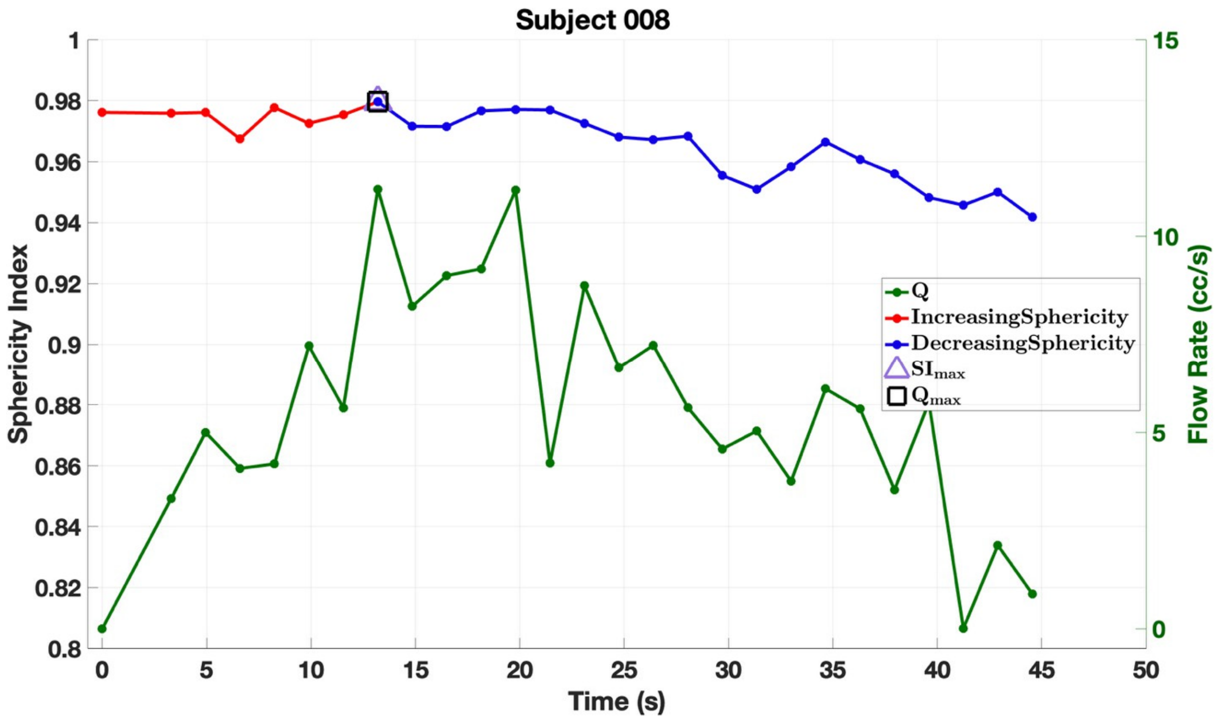

Subject 008
Sphericity Index
Flow Rate (cc/s)
Time (s)
Q
IncreasingSphericity
DecreasingSphericity
SI_{max}
Q_{max}

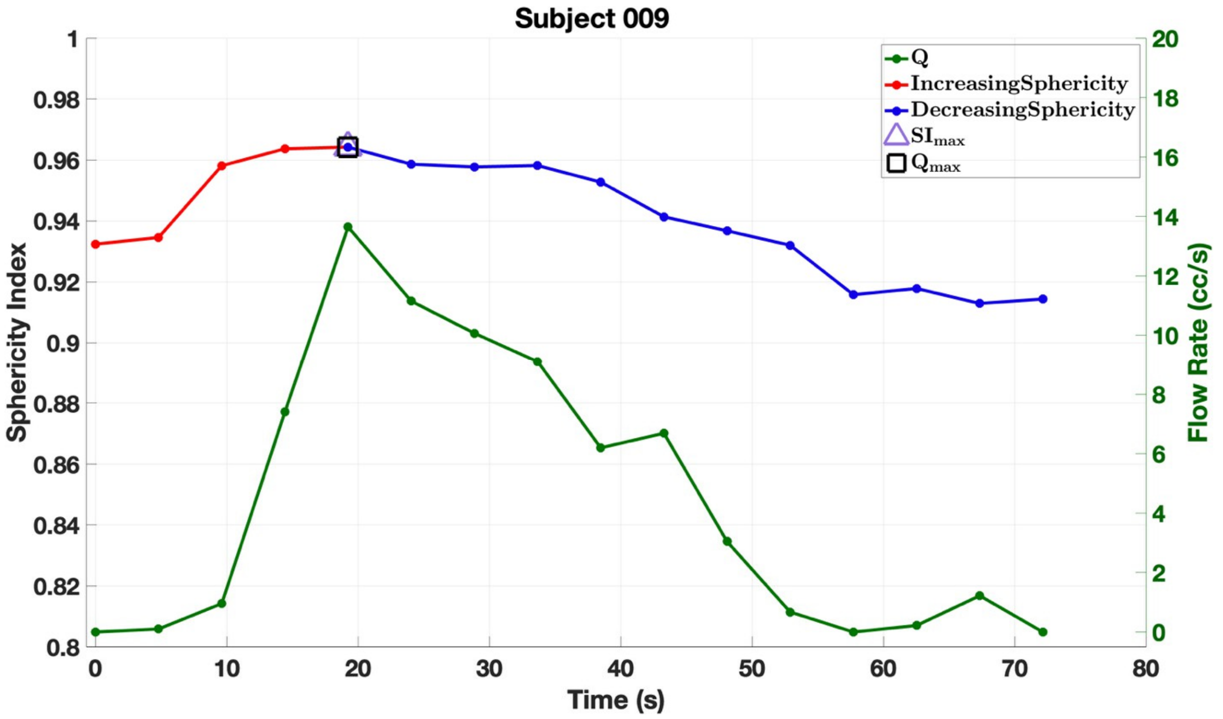

Subject 009
Sphericity Index
Flow Rate (cc/s)
Time (s)
Q
IncreasingSphericity
DecreasingSphericity
SI_{max}
Q_{max}

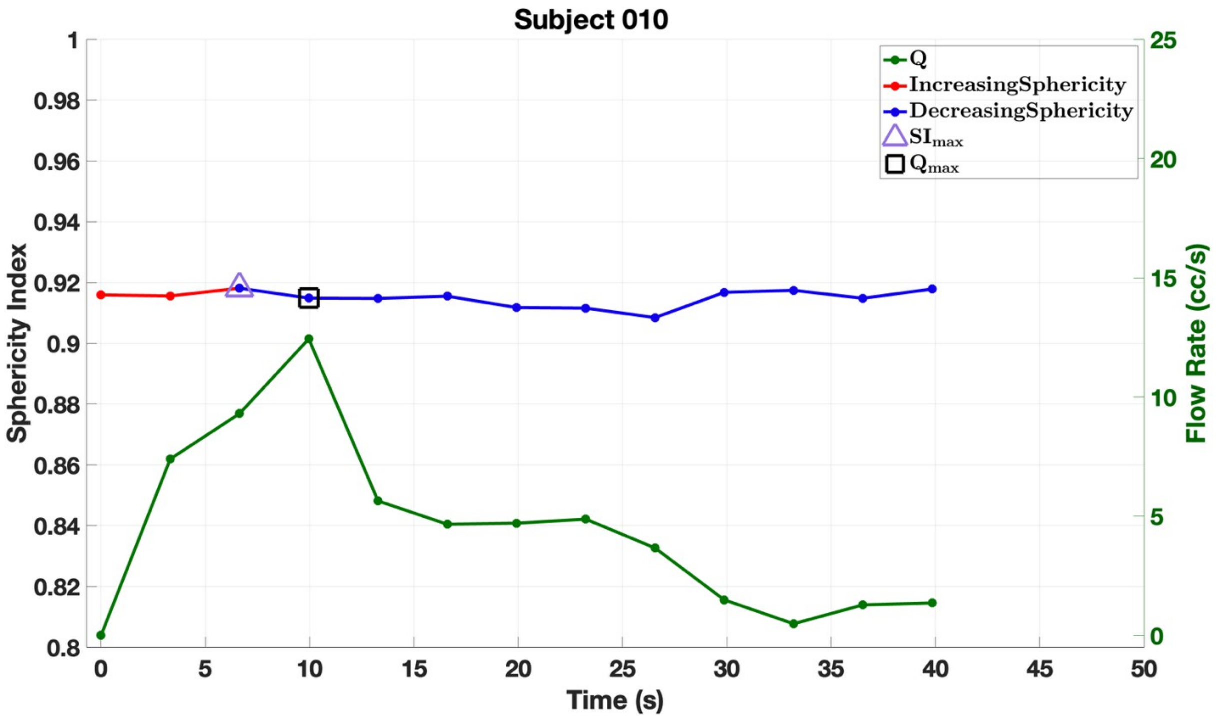
Subject 010
Sphericity Index
Flow Rate (cc/s)
Time (s)
Q
IncreasingSphericity
DecreasingSphericity
SI_{max}
Q_{max}
1
0.98
0.96
0.94
0.92
0.9
0.88
0.86
0.84
0.82
0.8
25
20
15
10
5
0
0
5
10
15
20
25
30
35
40
45
50

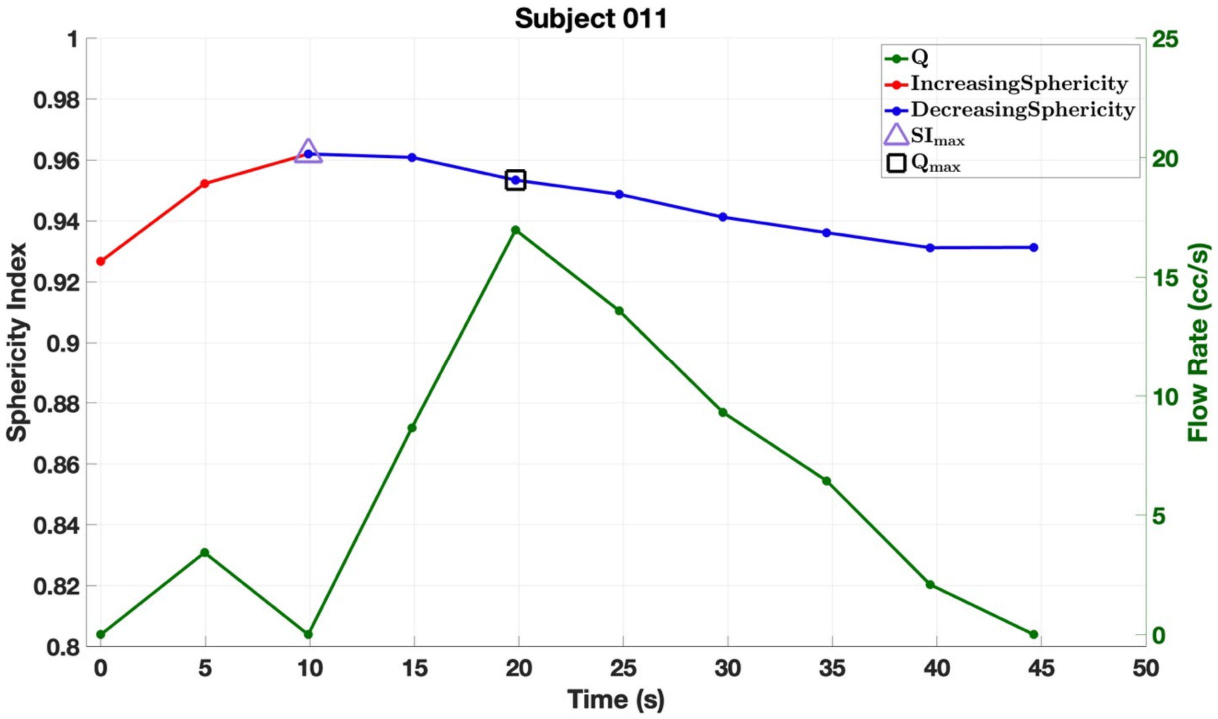
Subject 011
Sphericity Index
Flow Rate (cc/s)
Time (s)
Q
IncreasingSphericity
DecreasingSphericity
SI_{max}
Q_{max}
1
0.98
0.96
0.94
0.92
0.9
0.88
0.86
0.84
0.82
0.8
25
20
15
10
5
0
0
5
10
15
20
25
30
35
40
45
50

Supplementary Materials S2: Deformation Maps

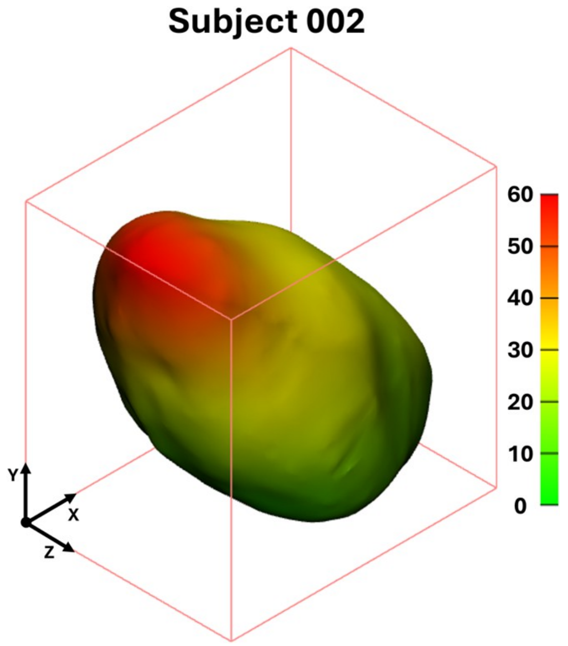

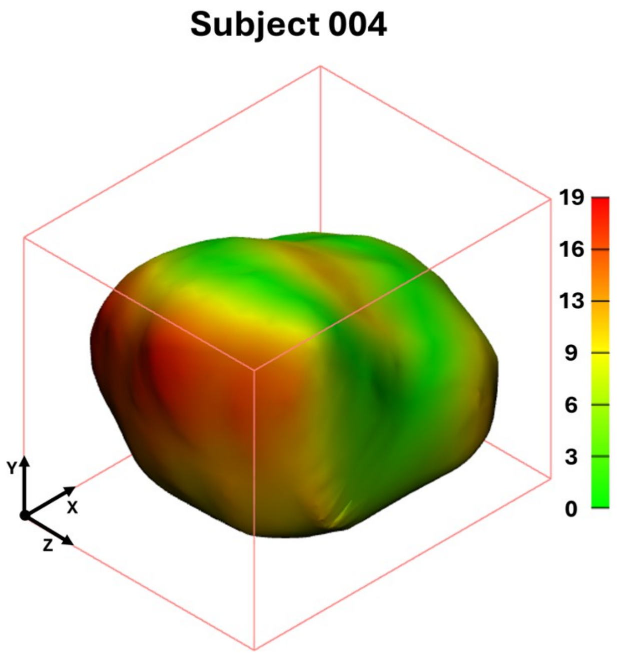

Subject 005

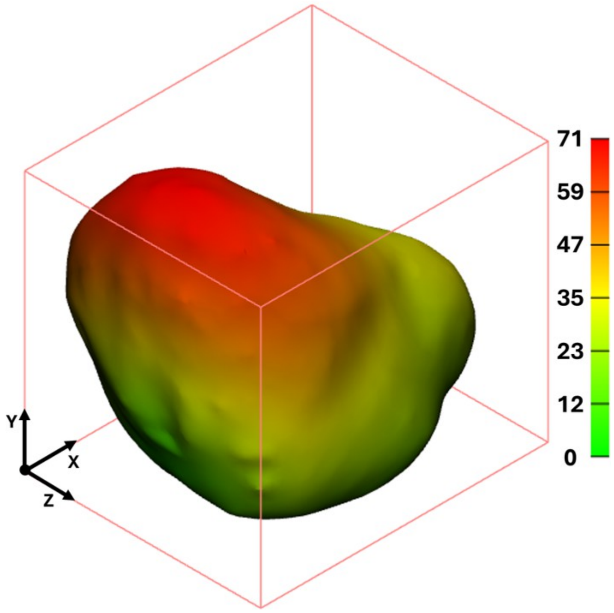

Subject 006

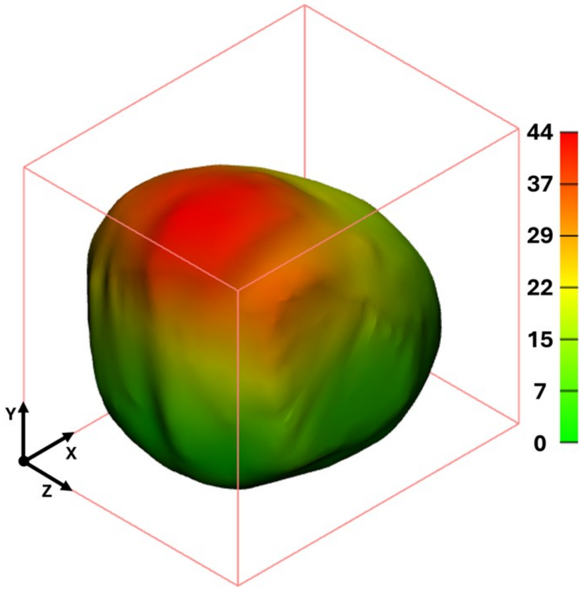

Subject 007

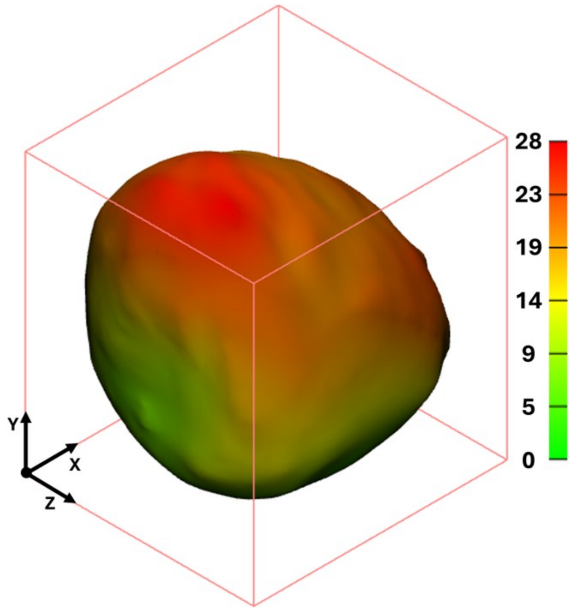

Subject 008

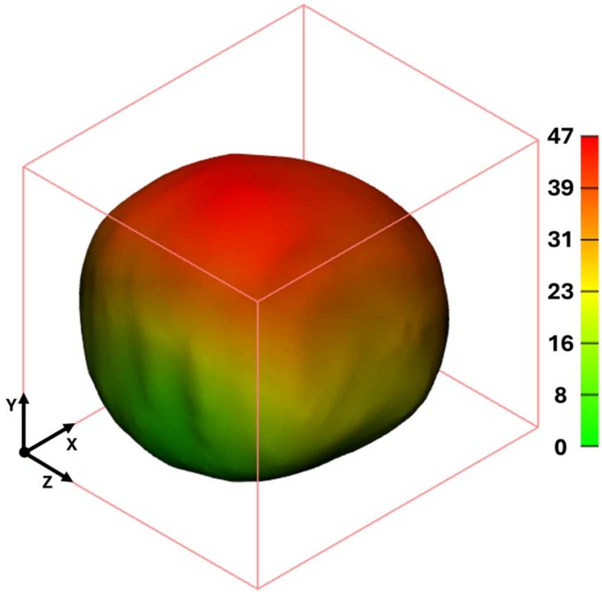

Subject 009

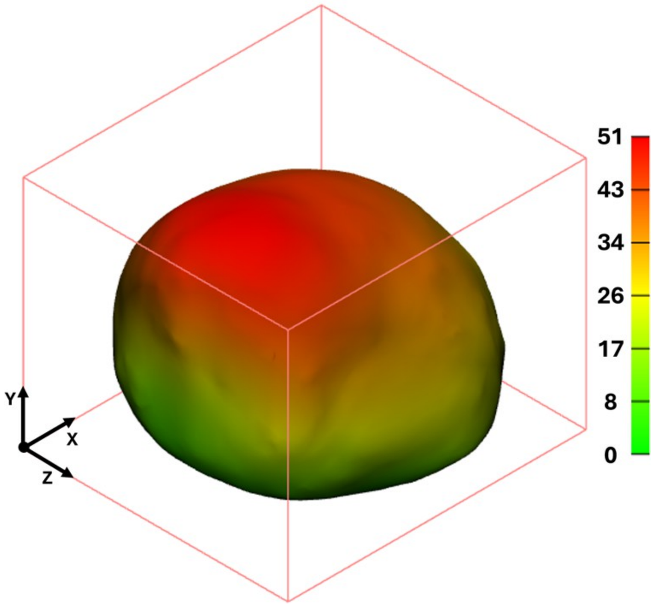

Subject 010

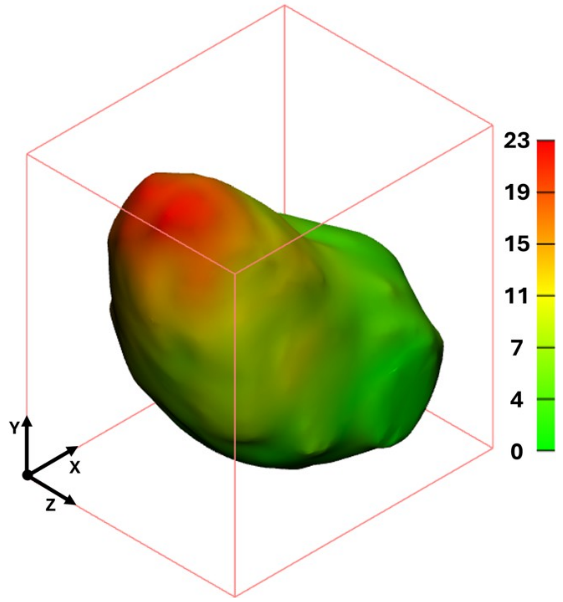

Subject 011

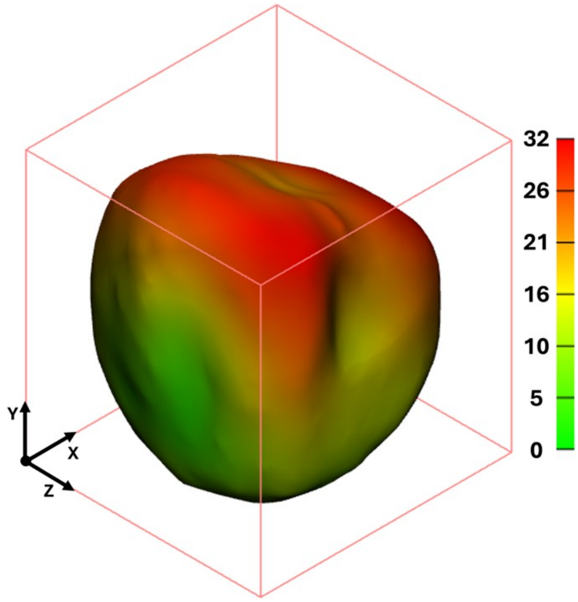

Supplementary Materials S3: Anatomical Measurements Correlated with Flow Rate

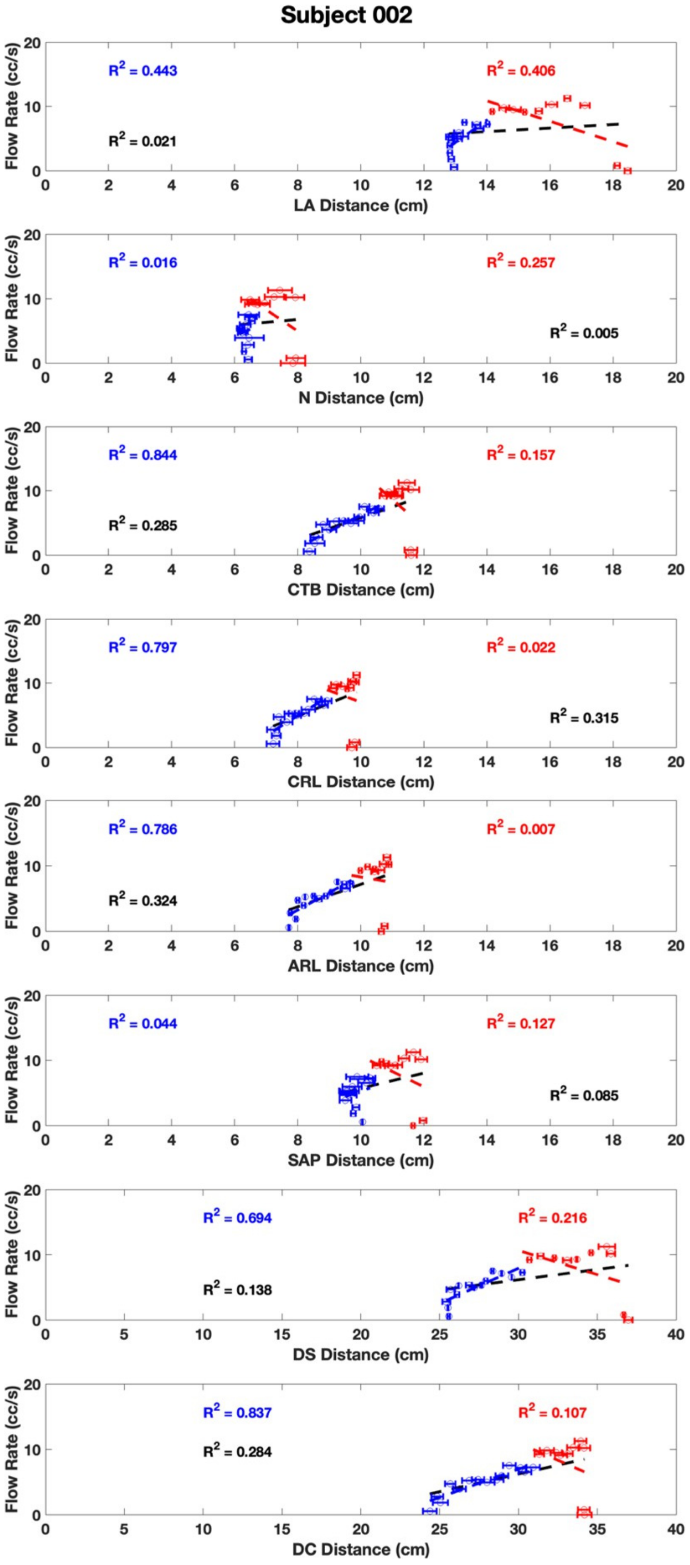

Subject 004

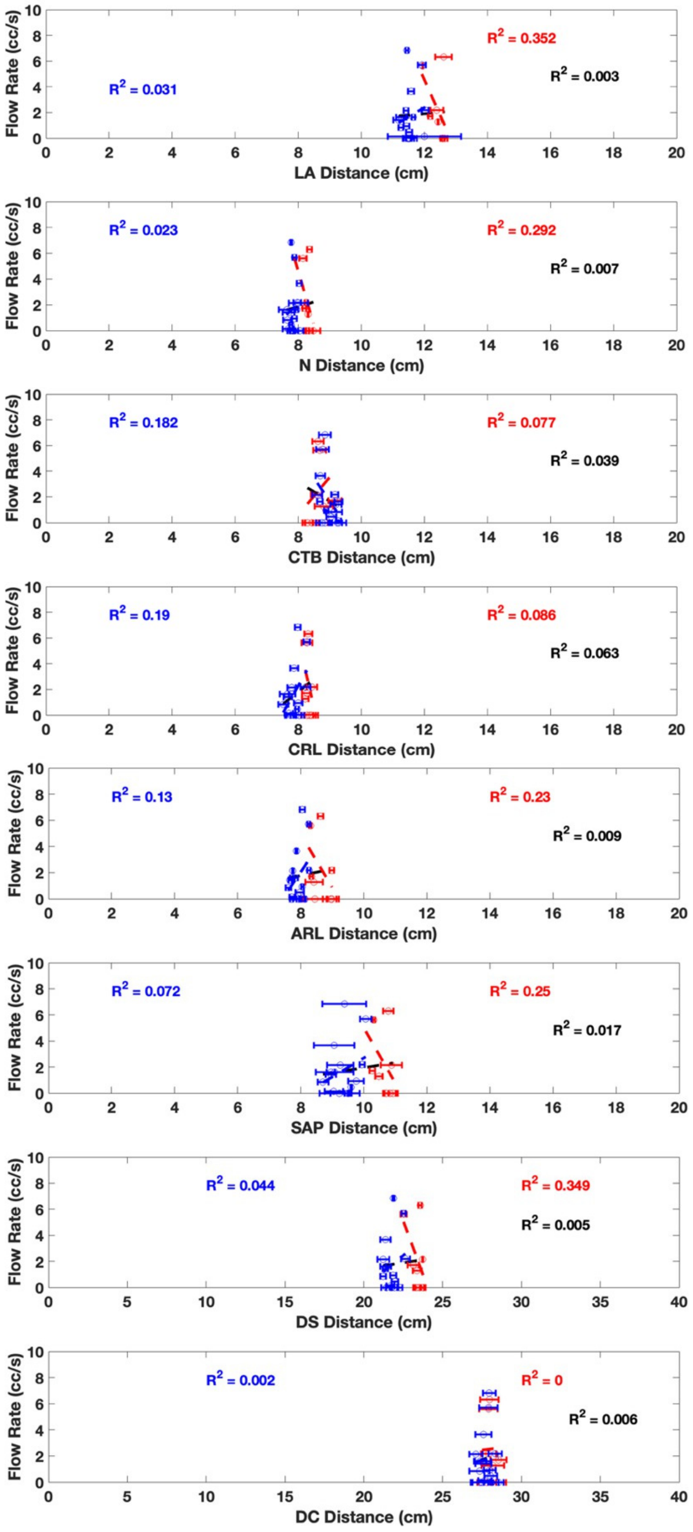

Flow Rate (cc/s)
LA Distance (cm)
R^2 = 0.031
R^2 = 0.352
R^2 = 0.003
N Distance (cm)
R^2 = 0.023
R^2 = 0.292
R^2 = 0.007
CTB Distance (cm)
R^2 = 0.182
R^2 = 0.077
R^2 = 0.039
CRL Distance (cm)
R^2 = 0.19
R^2 = 0.086
R^2 = 0.063
ARL Distance (cm)
R^2 = 0.13
R^2 = 0.23
R^2 = 0.009
SAP Distance (cm)
R^2 = 0.072
R^2 = 0.25
R^2 = 0.017
DS Distance (cm)
R^2 = 0.044
R^2 = 0.349
R^2 = 0.005
DC Distance (cm)
R^2 = 0.002
R^2 = 0
R^2 = 0.006

Subject 005

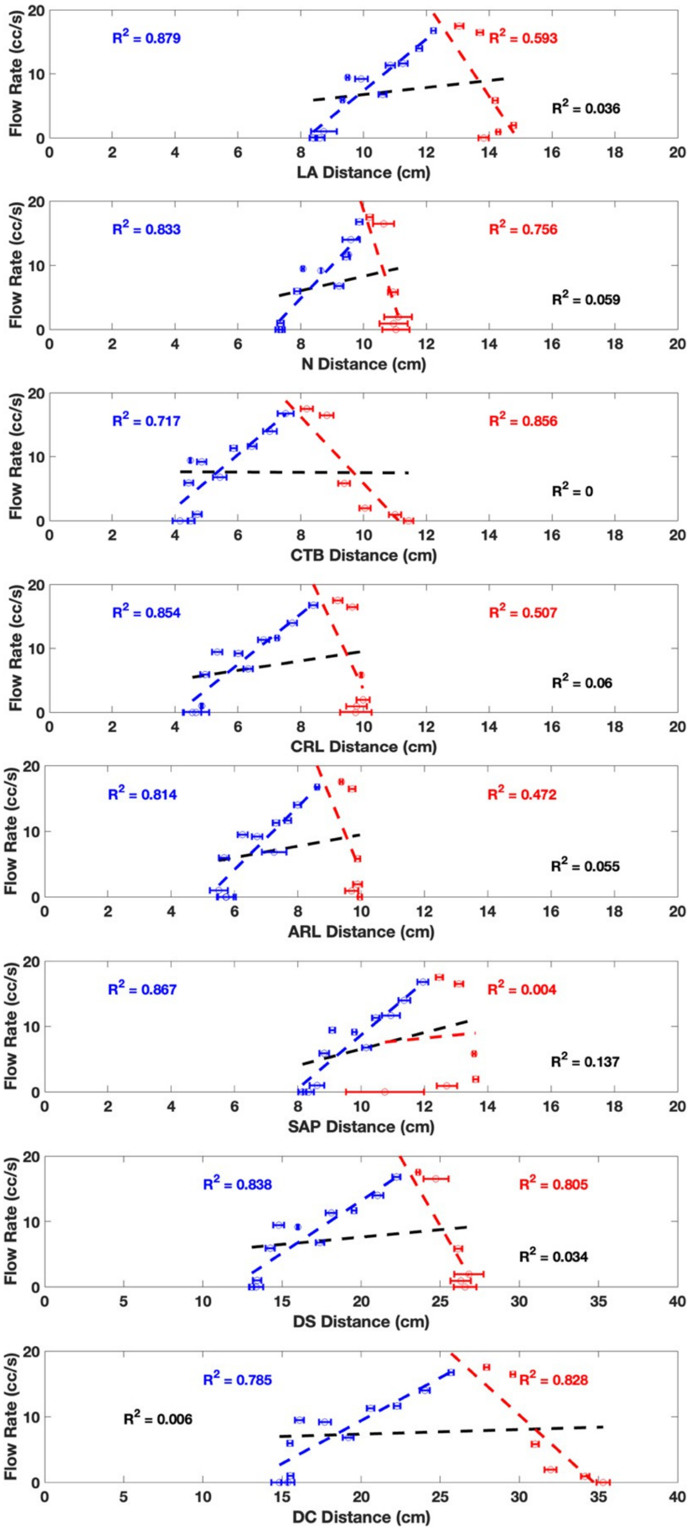

Flow Rate (cc/s)
LA Distance (cm)
R² = 0.879
R² = 0.593
R² = 0.036
N Distance (cm)
R² = 0.833
R² = 0.756
R² = 0.059
CTB Distance (cm)
R² = 0.717
R² = 0.856
R² = 0
CRL Distance (cm)
R² = 0.854
R² = 0.507
R² = 0.06
ARL Distance (cm)
R² = 0.814
R² = 0.472
R² = 0.055
SAP Distance (cm)
R² = 0.867
R² = 0.004
R² = 0.137
DS Distance (cm)
R² = 0.838
R² = 0.805
R² = 0.034
DC Distance (cm)
R² = 0.785
R² = 0.828
R² = 0.006

Subject 006

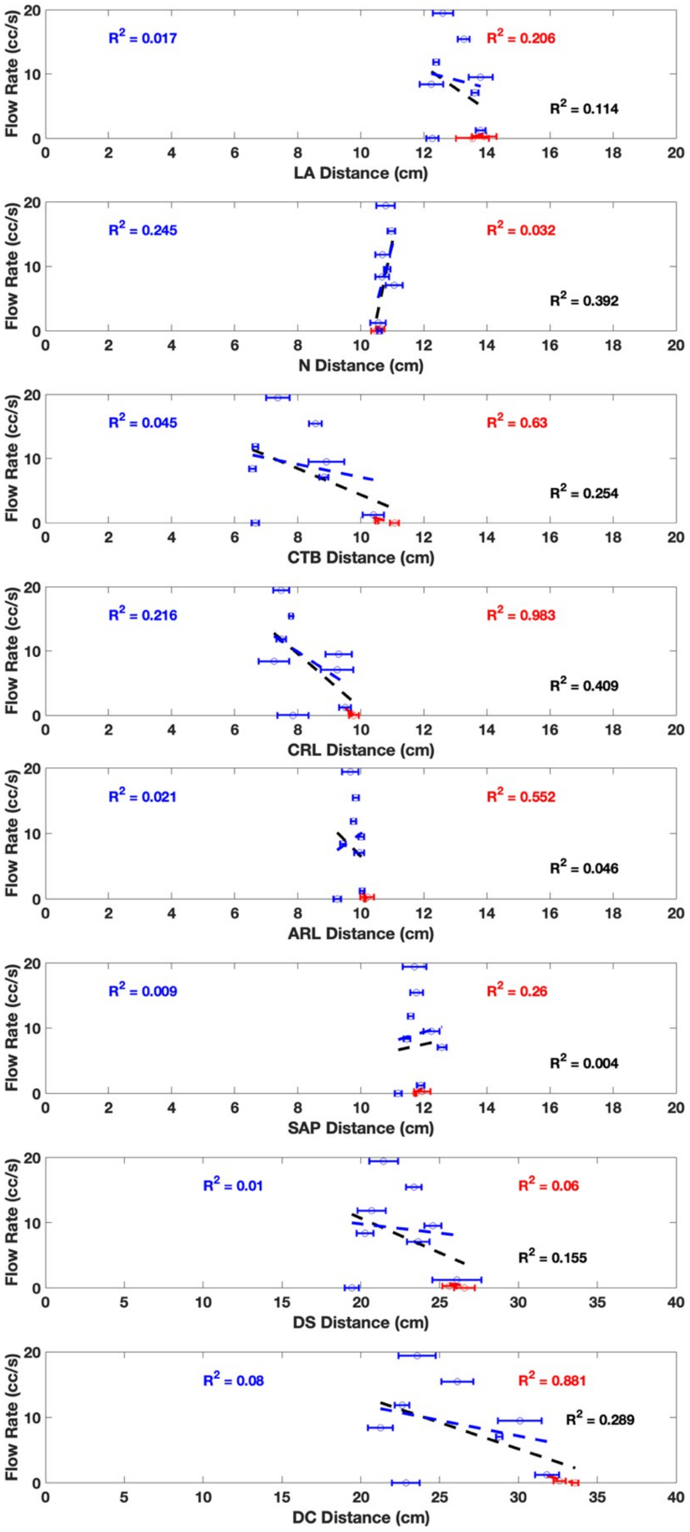

Flow Rate (cc/s)
LA Distance (cm)
N Distance (cm)
CTB Distance (cm)
CRL Distance (cm)
ARL Distance (cm)
SAP Distance (cm)
DS Distance (cm)
DC Distance (cm)
R2 = 0.017
R2 = 0.206
R2 = 0.114
R2 = 0.245
R2 = 0.032
R2 = 0.392
R2 = 0.045
R2 = 0.63
R2 = 0.254
R2 = 0.216
R2 = 0.983
R2 = 0.409
R2 = 0.021
R2 = 0.552
R2 = 0.046
R2 = 0.009
R2 = 0.26
R2 = 0.004
R2 = 0.01
R2 = 0.06
R2 = 0.155
R2 = 0.08
R2 = 0.881
R2 = 0.289

Subject 007

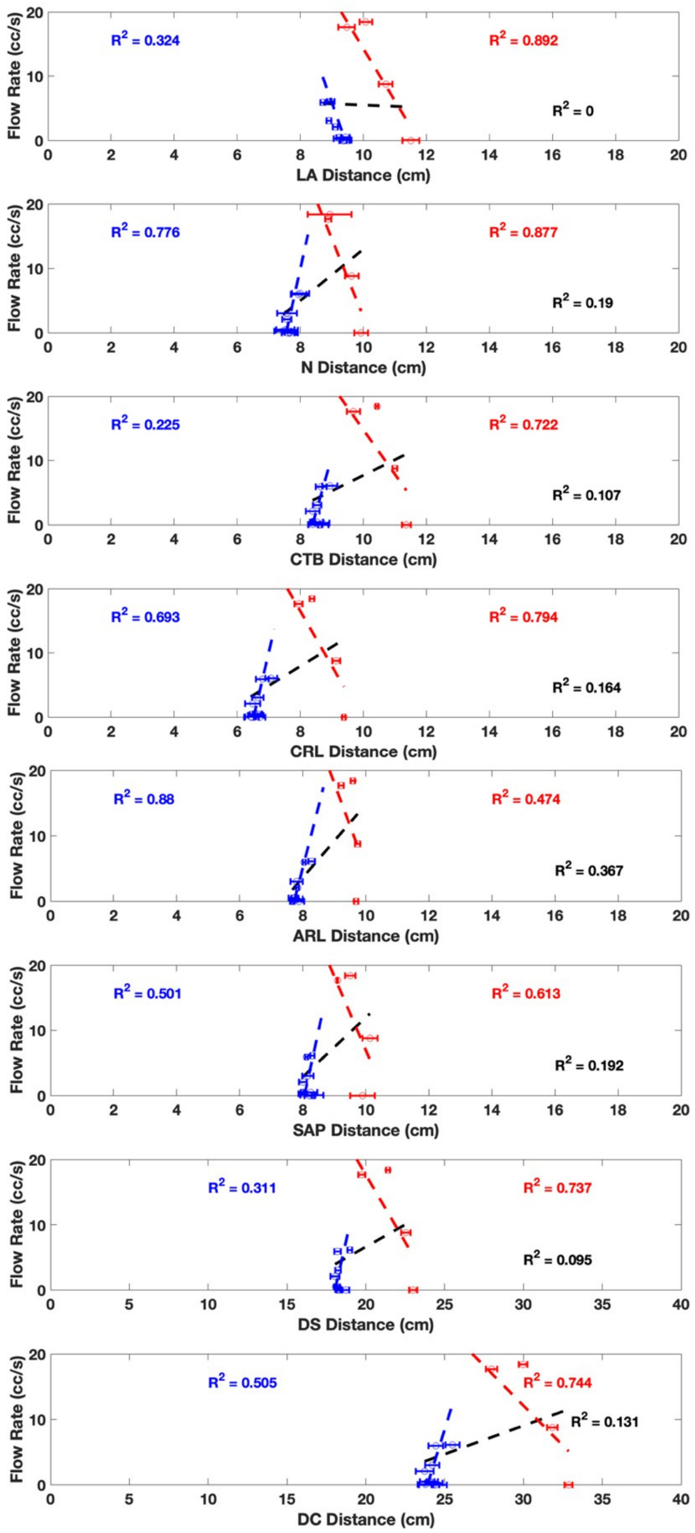

Flow Rate (cc/s)
R^2 = 0.324
R^2 = 0.892
R^2 = 0
LA Distance (cm)
R^2 = 0.776
R^2 = 0.877
R^2 = 0.19
N Distance (cm)
R^2 = 0.225
R^2 = 0.722
R^2 = 0.107
CTB Distance (cm)
R^2 = 0.693
R^2 = 0.794
R^2 = 0.164
CRL Distance (cm)
R^2 = 0.88
R^2 = 0.474
R^2 = 0.367
ARL Distance (cm)
R^2 = 0.501
R^2 = 0.613
R^2 = 0.192
SAP Distance (cm)
R^2 = 0.311
R^2 = 0.737
R^2 = 0.095
DS Distance (cm)
R^2 = 0.505
R^2 = 0.744
R^2 = 0.131
DC Distance (cm)

Subject 008

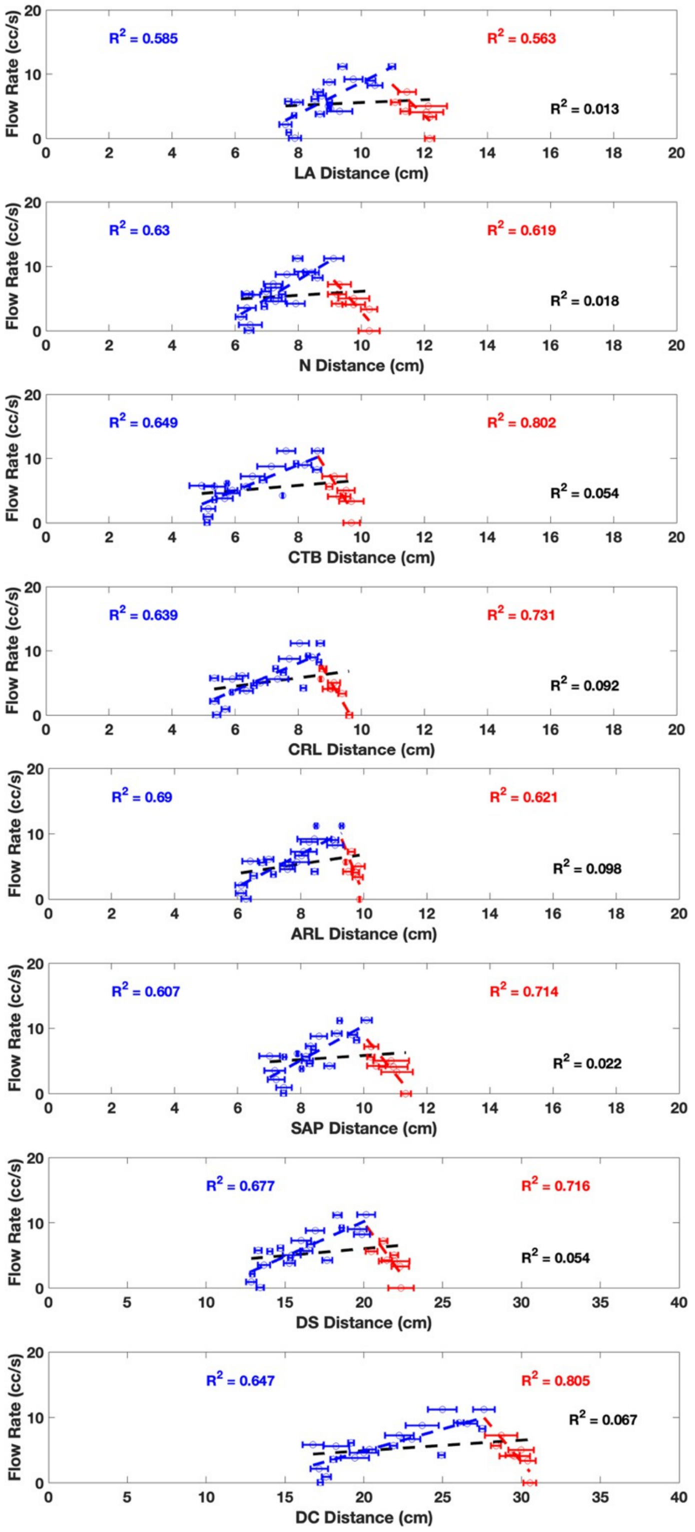

Flow Rate (cc/s)
R² = 0.585
R² = 0.563
R² = 0.013
LA Distance (cm)
R² = 0.63
R² = 0.619
R² = 0.018
N Distance (cm)
R² = 0.649
R² = 0.802
R² = 0.054
CTB Distance (cm)
R² = 0.639
R² = 0.731
R² = 0.092
CRL Distance (cm)
R² = 0.69
R² = 0.621
R² = 0.098
ARL Distance (cm)
R² = 0.607
R² = 0.714
R² = 0.022
SAP Distance (cm)
R² = 0.677
R² = 0.716
R² = 0.054
DS Distance (cm)
R² = 0.647
R² = 0.805
R² = 0.067
DC Distance (cm)

Subject 009

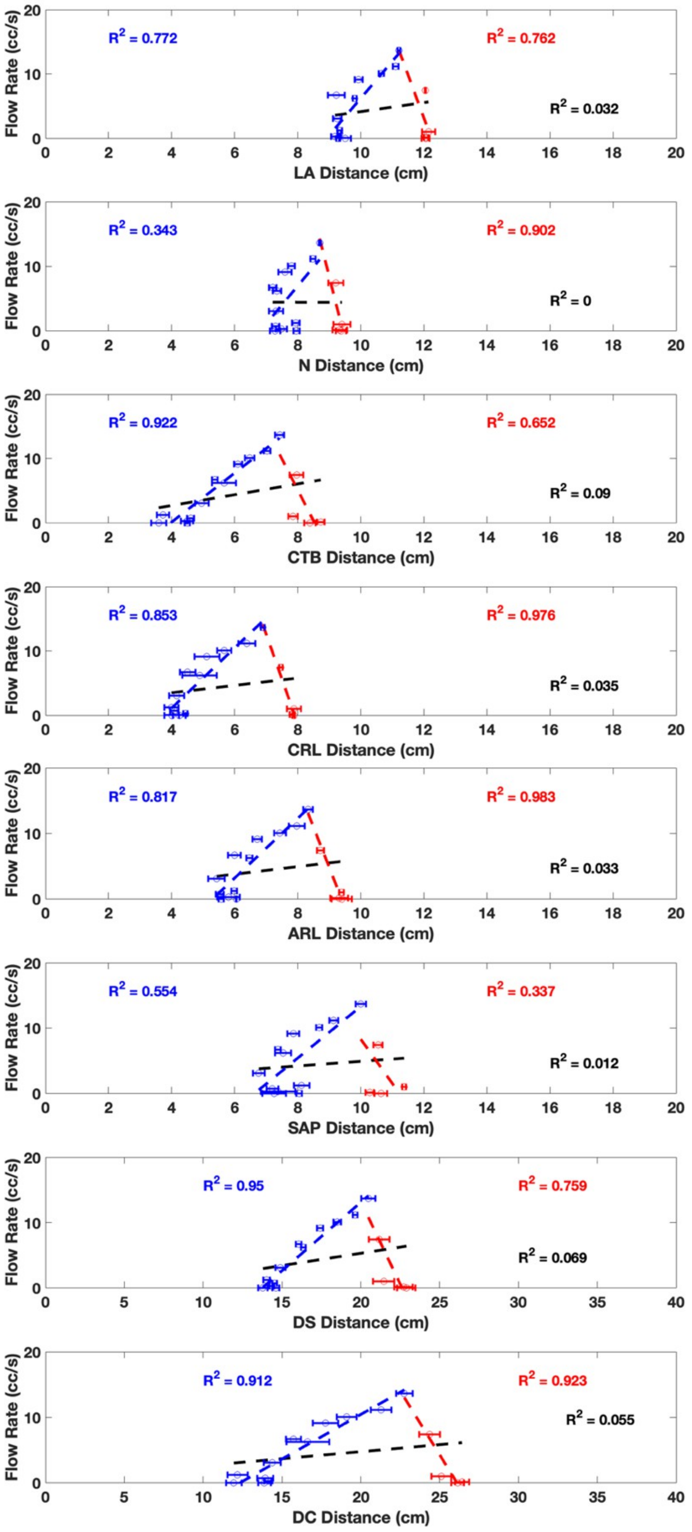

Flow Rate (cc/s)
R² = 0.772
R² = 0.762
R² = 0.032
LA Distance (cm)
R² = 0.343
R² = 0.902
R² = 0
N Distance (cm)
R² = 0.922
R² = 0.652
R² = 0.09
CTB Distance (cm)
R² = 0.853
R² = 0.976
R² = 0.035
CRL Distance (cm)
R² = 0.817
R² = 0.983
R² = 0.033
ARL Distance (cm)
R² = 0.554
R² = 0.337
R² = 0.012
SAP Distance (cm)
R² = 0.95
R² = 0.759
R² = 0.069
DS Distance (cm)
R² = 0.912
R² = 0.923
R² = 0.055
DC Distance (cm)

Subject 010

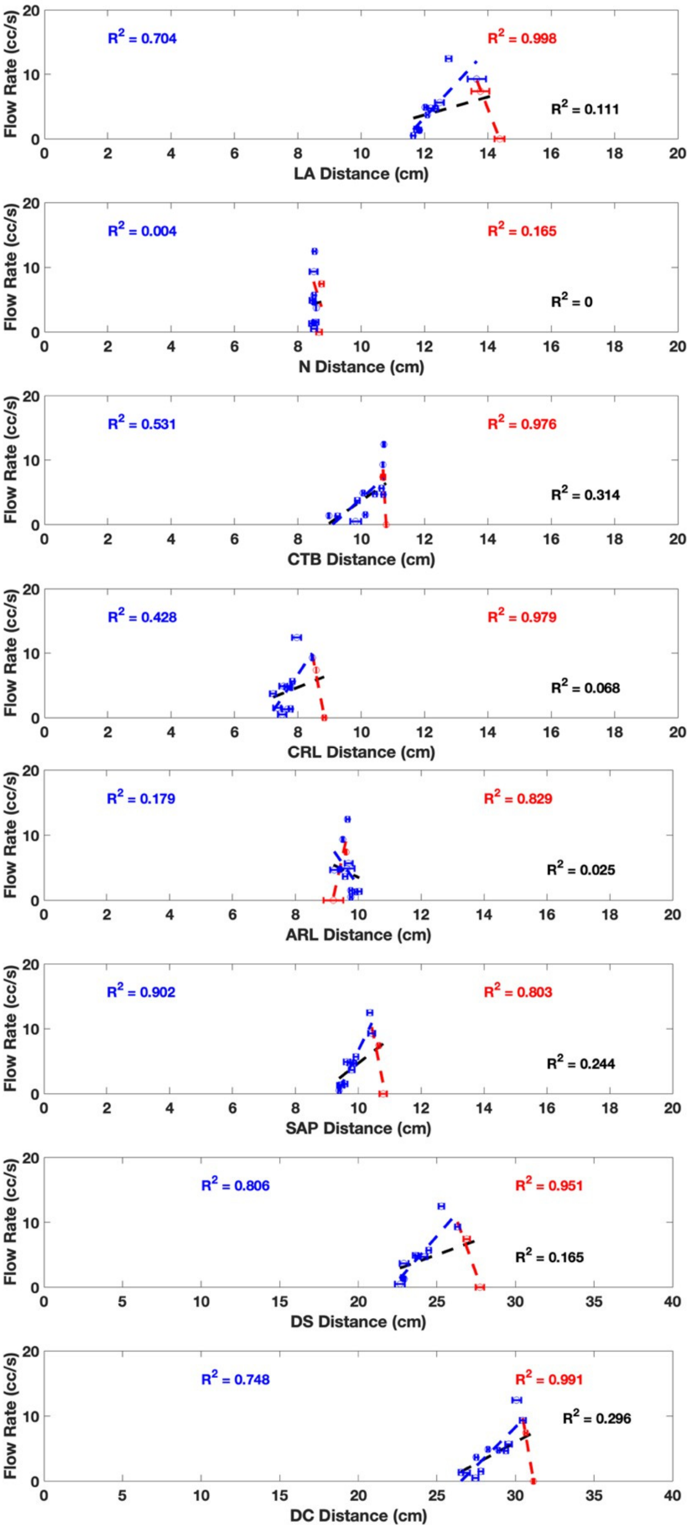

Flow Rate (cc/s)
R2 = 0.704
R2 = 0.998
R2 = 0.111
LA Distance (cm)
R2 = 0.004
R2 = 0.165
R2 = 0
N Distance (cm)
R2 = 0.531
R2 = 0.976
R2 = 0.314
CTB Distance (cm)
R2 = 0.428
R2 = 0.979
R2 = 0.068
CRL Distance (cm)
R2 = 0.179
R2 = 0.829
R2 = 0.025
ARL Distance (cm)
R2 = 0.902
R2 = 0.803
R2 = 0.244
SAP Distance (cm)
R2 = 0.806
R2 = 0.951
R2 = 0.165
DS Distance (cm)
R2 = 0.748
R2 = 0.991
R2 = 0.296
DC Distance (cm)

Subject 011

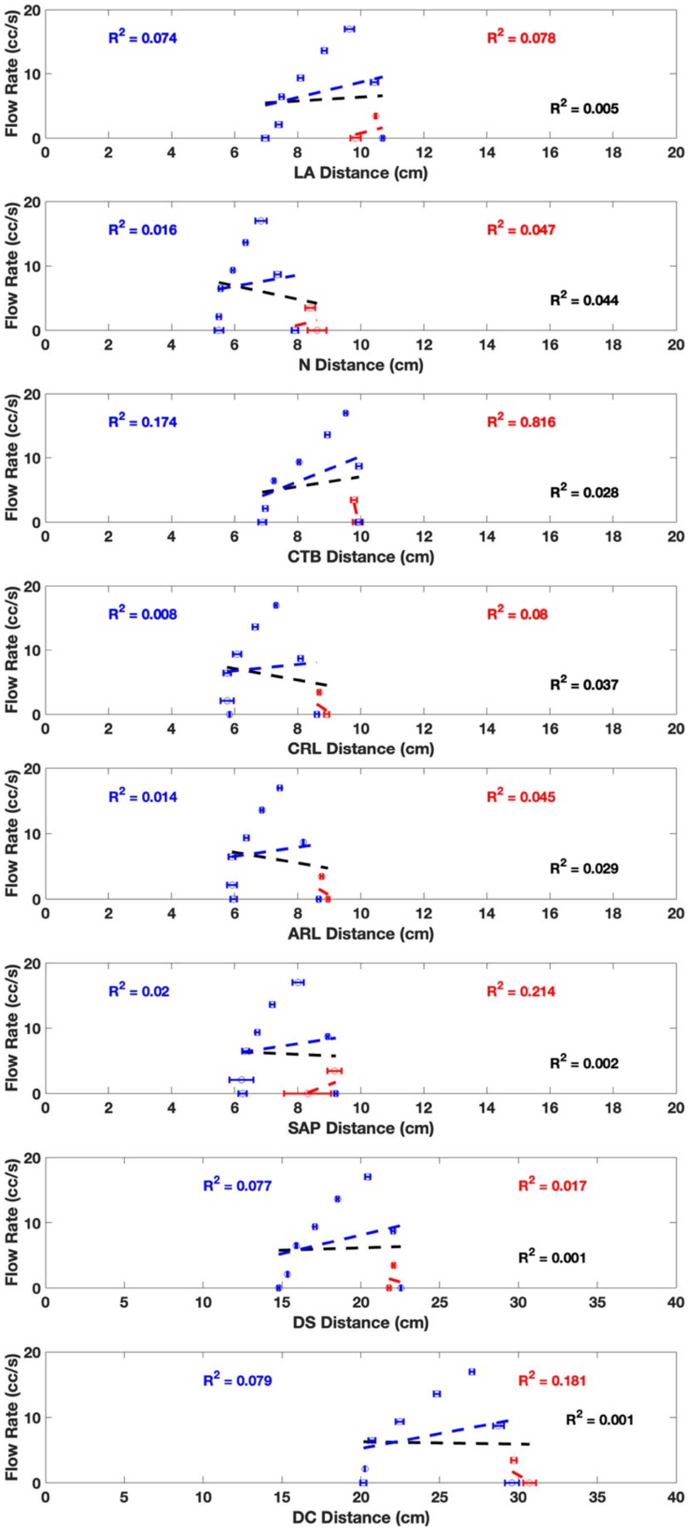

Flow Rate (cc/s)
R² = 0.074
R² = 0.078
R² = 0.005
LA Distance (cm)
R² = 0.016
R² = 0.047
R² = 0.044
N Distance (cm)
R² = 0.174
R² = 0.816
R² = 0.028
CTB Distance (cm)
R² = 0.008
R² = 0.08
R² = 0.037
CRL Distance (cm)
R² = 0.014
R² = 0.045
R² = 0.029
ARL Distance (cm)
R² = 0.02
R² = 0.214
R² = 0.002
SAP Distance (cm)
R² = 0.077
R² = 0.017
R² = 0.001
DS Distance (cm)
R² = 0.079
R² = 0.181
R² = 0.001
DC Distance (cm)

Supplementary Materials S4: Rotation and Contraction; Frequency Decompositions

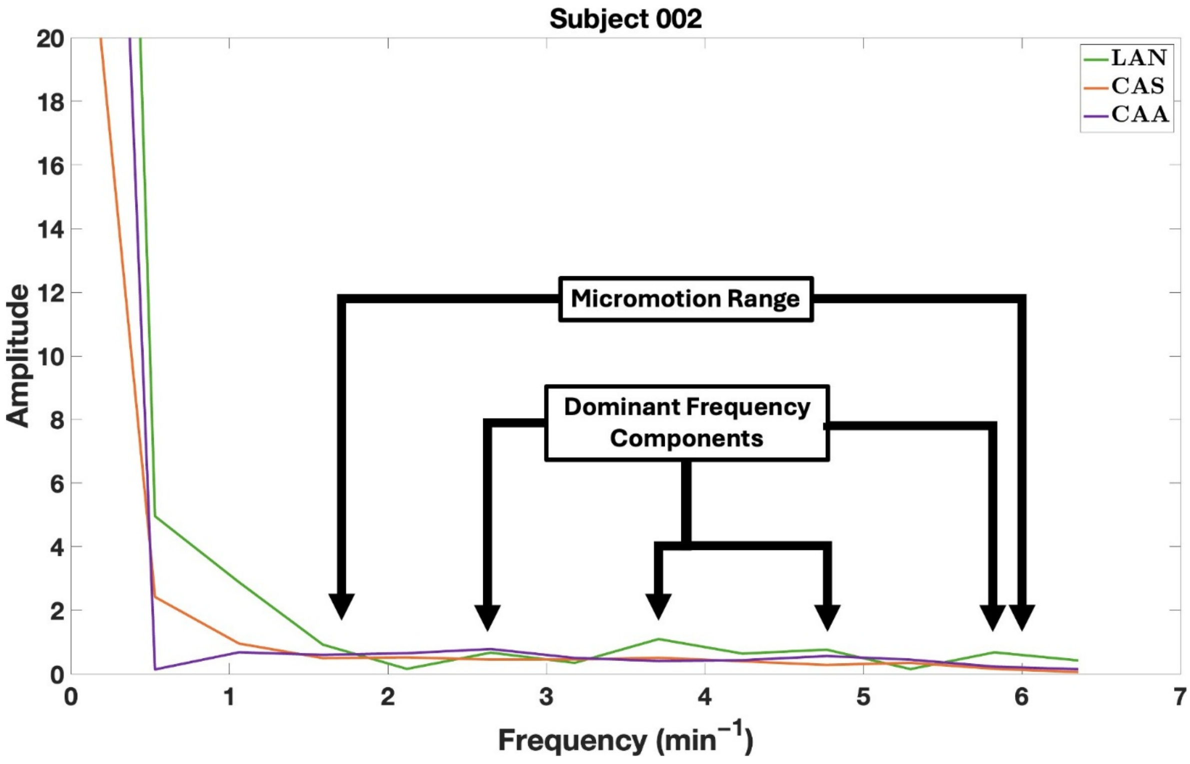

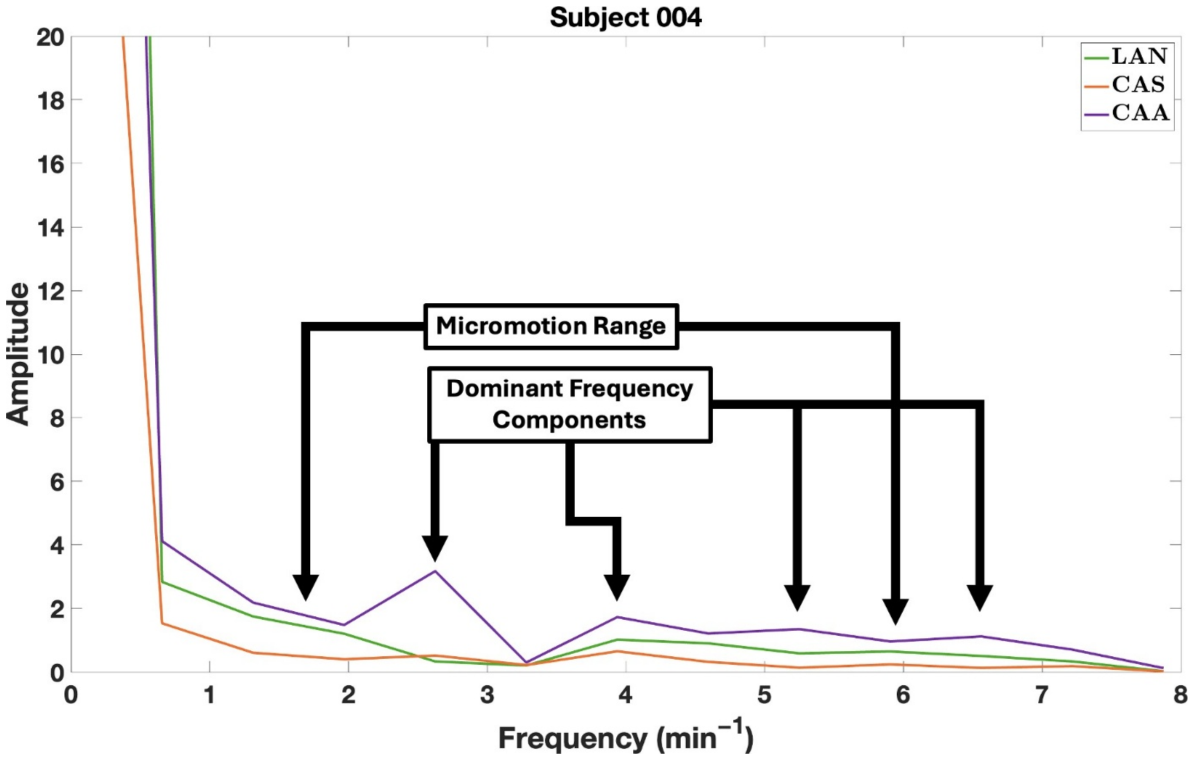

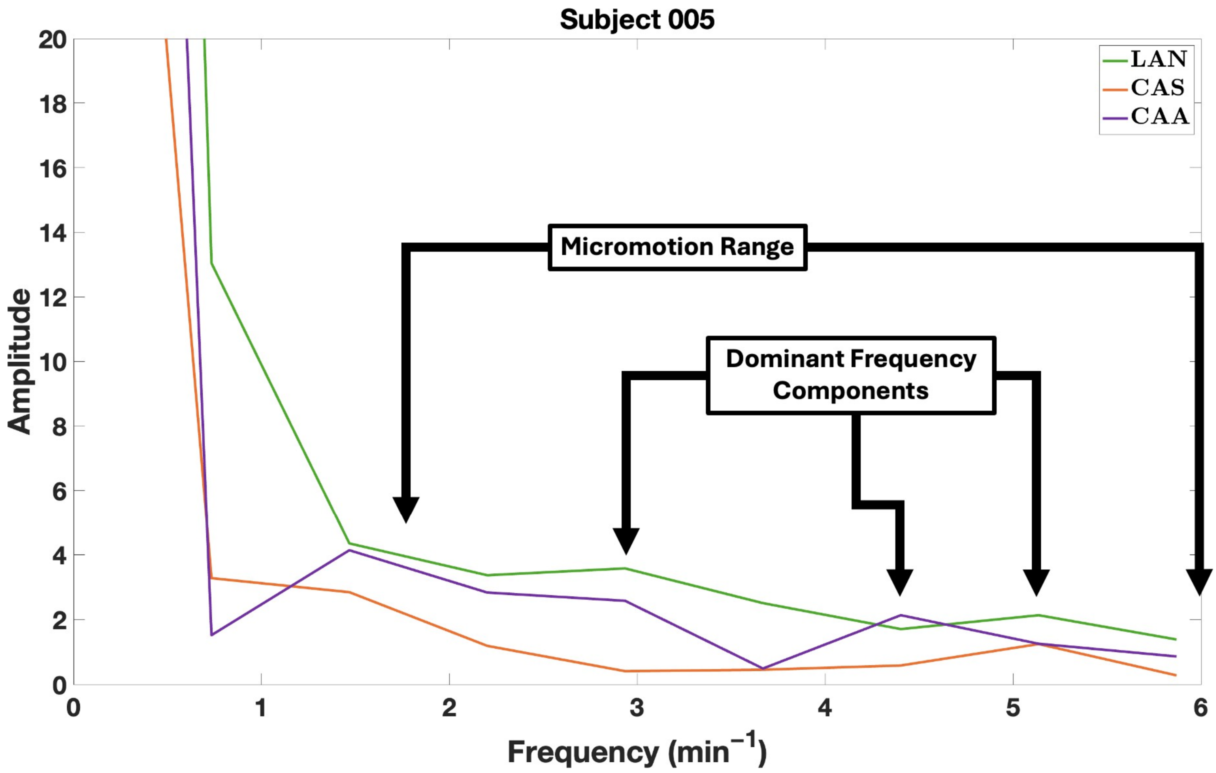
Subject 005
LAN
CAS
CAA
Micromotion Range
Dominant Frequency Components
Amplitude
Frequency (min^{-1})
0
2
4
6
8
10
12
14
16
18
20
1
3
5

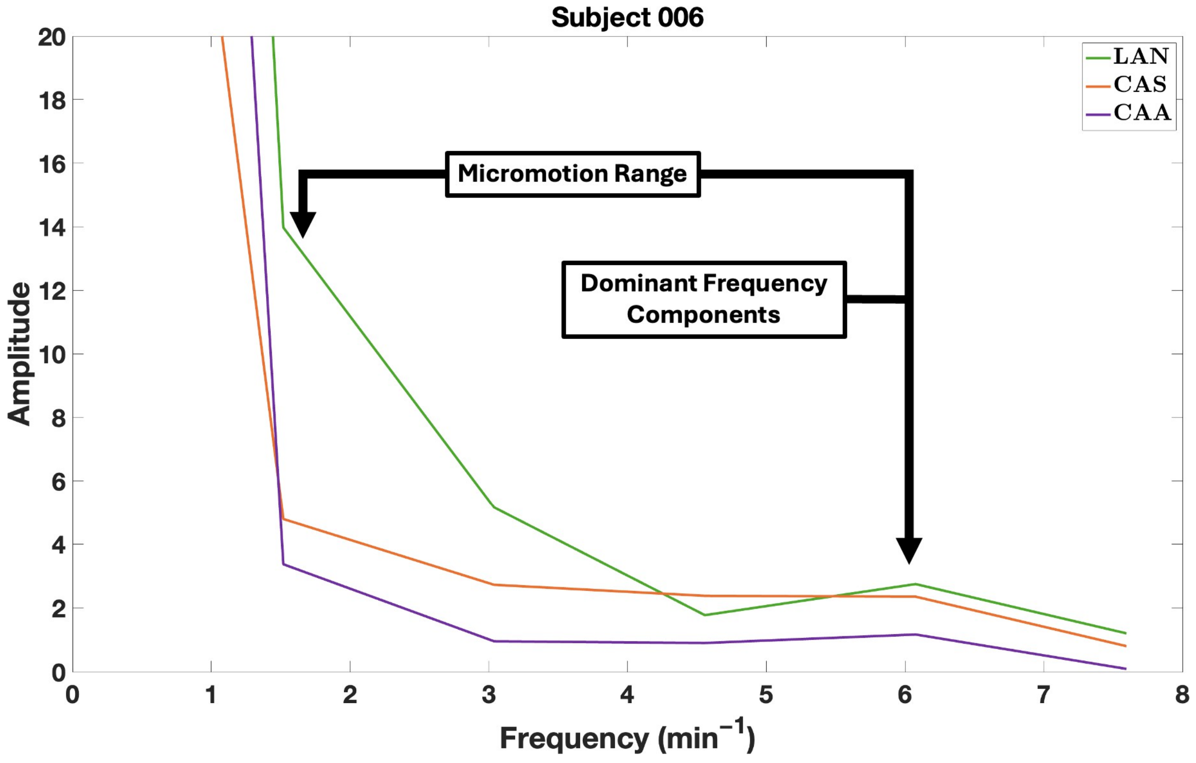
Subject 006
LAN
CAS
CAA
Micromotion Range
Dominant Frequency Components
Amplitude
Frequency (min^{-1})
0
2
4
6
8
10
12
14
16
18
20
1
3
5
7

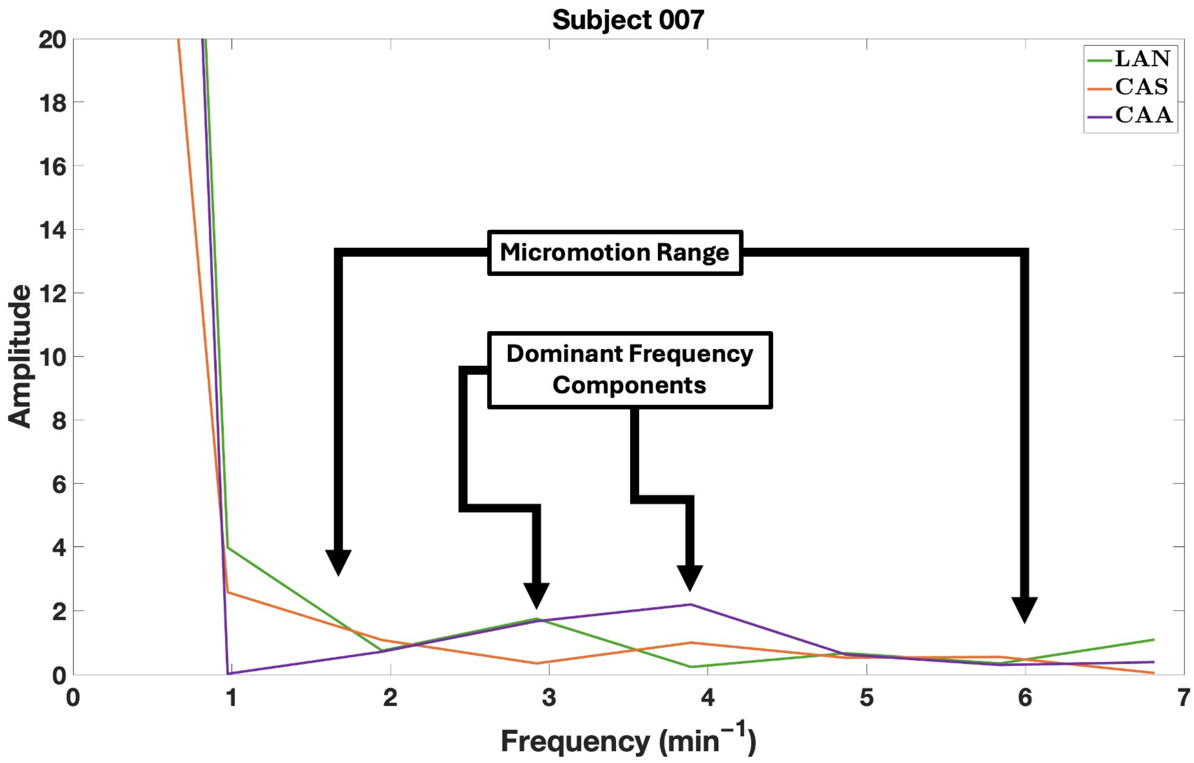
Subject 007
Amplitude
20
18
16
14
12
10
8
6
4
2
0
0
1
2
3
4
5
6
7
Frequency (min^{-1})
LAN
CAS
CAA
Micromotion Range
Dominant Frequency Components

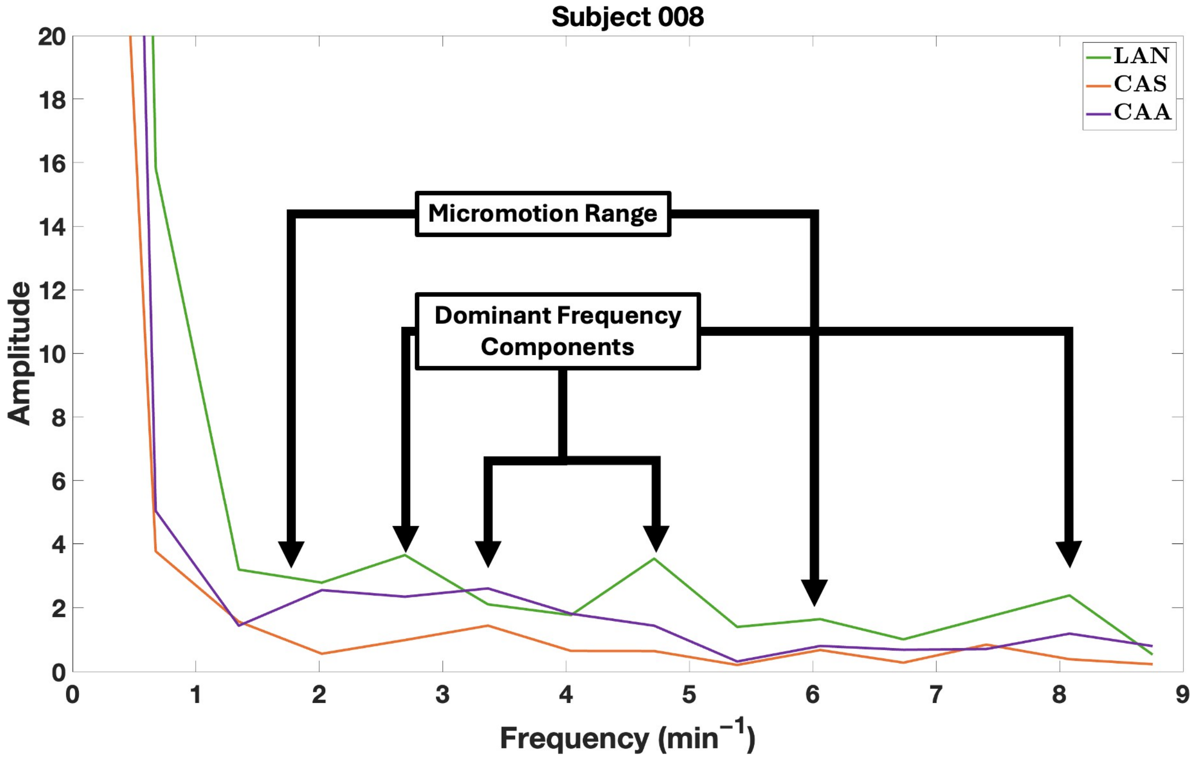
Subject 008
Amplitude
20
18
16
14
12
10
8
6
4
2
0
0
1
2
3
4
5
6
7
8
9
Frequency (min^{-1})
LAN
CAS
CAA
Micromotion Range
Dominant Frequency Components

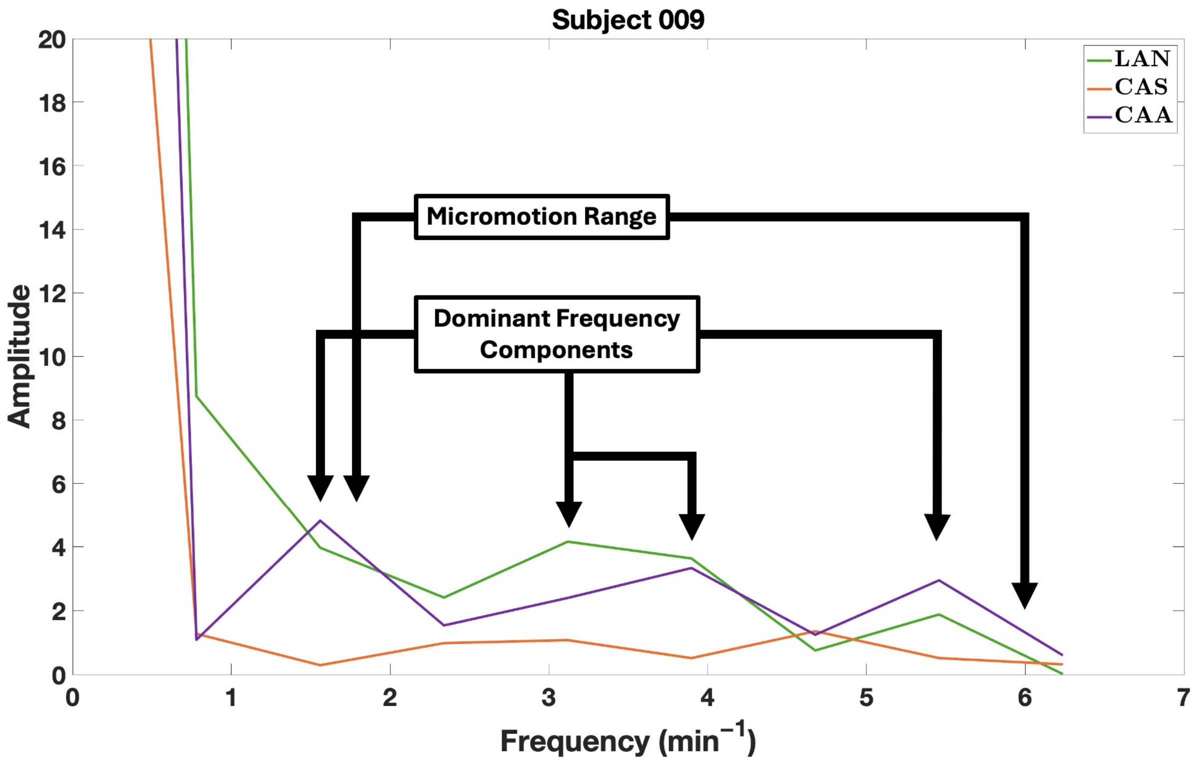
Subject 009
Amplitude
Frequency (min^{-1})
LAN
CAS
CAA
Micromotion Range
Dominant Frequency Components

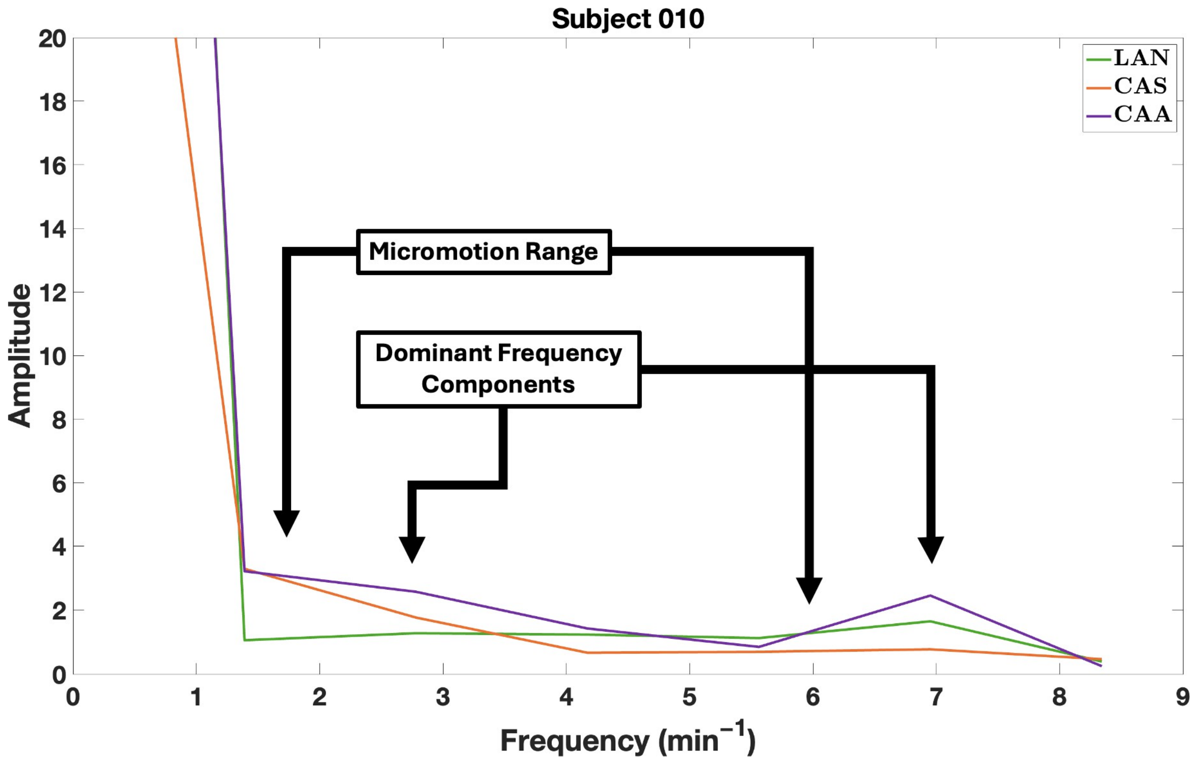
Subject 010
Amplitude
Frequency (min^{-1})
LAN
CAS
CAA
Micromotion Range
Dominant Frequency Components

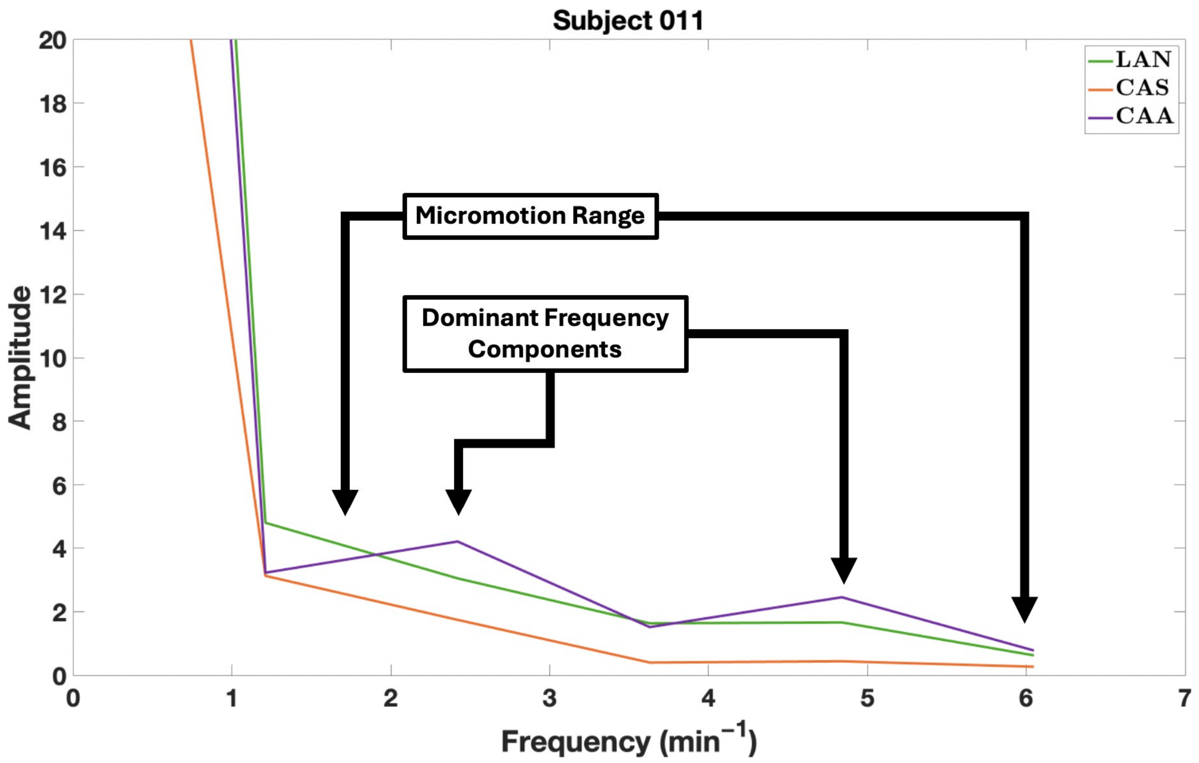

Subject 011
20
18
16
14
12
10
8
6
4
2
0
Amplitude
0
1
2
3
4
5
6
7
Frequency (min^{-1})
LAN
CAS
CAA
Micromotion Range
Dominant Frequency
Components
